# Macrophage migration inhibitory factor contributes to the pathogenesis of benign lymphoepithelial lesion of the lacrimal gland

**DOI:** 10.1186/s12964-018-0284-4

**Published:** 2018-10-22

**Authors:** Yao Mawulikplimi Adzavon, Pengxiang Zhao, Jianmin Ma, Xujuan Zhang, Xin Zhang, Mingzi Zhang, Mengyu Liu, Limin Wang, Danying Chen, Tarekegn Gebreyesus Abisso, Baobei Lv, Lei Wang, Fei Xie, Xuemei Ma

**Affiliations:** 1 College of Life Science and Bio-engineering, Beijing Molecular Hydrogen Research Center, Beijing University of Technology, Beijing, 100124 People’s Republic of China; 2 Beijing Tongren Hospital, Capital Medical University, Beijing, 100730 People’s Republic of China; 3 Beijing Ophthalmology & Vision Science Key Lab, Beijing Tongren Eye Center, Beijing, 100730 People’s Republic of China; 4 Department of Plastic Surgery, Peking Union Medical College Hospital, Beijing, 100730 People’s Republic of China; 5 Beijing Ditan Hospital, Capital Medical University, Beijing, 100015 People’s Republic of China

**Keywords:** Benign Lymphoepithelial lesion, Macrophage migration inhibitory factor, Pathogenesis, Inflammation, Fibrosis

## Abstract

**Background:**

Benign Lymphoepithelial Lesion (BLEL) is a rare disease observed in the adult population. Despite the growing numbers of people suffering from BLEL, the etiology and mechanisms underlying its pathogenesis remain unknown.

**Methods:**

In the present study, we used gene and cytokines expression profiling, western blot and immunohistochemistry to get further insight into the cellular and molecular mechanisms involved in the pathogenesis of BLEL of the lacrimal gland.

**Results:**

The results showed that Macrophage Migration Inhibitory Factor (MIF) was the most highly expressed cytokine in BLEL, and its expression positively correlated with the expression of Th2 and Th17 cells cytokines. MIF was found to regulate biological functions and pathways involved in BLEL pathogenesis, such as proliferation, resistance to apoptosis, MAPK and PI3K/Akt pathways. We also found that MIF promotes fibrosis in BLEL by inducing BLEL fibroblast differentiation into myofibroblasts as well as the synthesis and the deposit of extracellular matrix in BLEL tissues.

**Conclusions:**

Our findings demonstrate the contribution of MIF to the pathogenesis of BLEL of the lacrimal gland and suggested MIF as a promising therapeutic target for its treatment.

**Electronic supplementary material:**

The online version of this article (10.1186/s12964-018-0284-4) contains supplementary material, which is available to authorized users.

## Background

Benign lymphoepithelial lesion (BLEL), also referred to as Mikulicz disease, is characterized by the bilateral swelling of salivary and lacrimal glands and immunoglobulin G4 (IgG4)-positive plasma cell infiltration in the affected tissues [1, 2]. The first case of this disease was reported by Johann Mikulicz in 1888, and for decades, it was mistaken for Sjögren’s syndrome due to their histological similarities. However, based on clear clinical differences between these two conditions, Mikulicz disease has finally been established as a new clinical entity [1, 3]. Recently, Mikulicz disease was included among the IgG4-related diseases, which are recognized immune-mediated conditions due to elevated serum IgG4 levels and infiltration of abundant IgG4-bearing plasmacytes into affected tissues [1, 2, 4]. The exact cause of BLEL and the mechanisms underlying its pathogenesis remain unknown. However, it is thought to be a systemic inflammatory disorder mediated by type 2 helper (Th2) cells and regulatory immune reactions [5, 6]. BLEL mainly occurs in adult females and is histologically characterized by lymphocytic infiltration, low frequency of apoptosis [1–4], and different degrees of fibrosis [4, 7, 8]. Several cases of BLEL complicated by malignant lymphoma have been reported, suggesting a high risk of malignant transformation in patients with this disease [9–12]. Therefore, it is important to identify therapeutic targets for the treatment of this disease.

Macrophage migration inhibitory factor (MIF) is a pleiotropic, pro-inflammatory cytokine that has been implicated in the pathogenesis of several inflammatory disorders, autoimmune diseases, and tumors [13–15]. MIF promotes cell proliferation and migration by interacting with its proposed receptors cluster of differentiation 74 (CD74), C-X-C chemokine receptor 2 (CXCR2), CXCR4, and CXCR7 [13, 16–19], which may trigger the activation of several pathways, including mitogen-activated protein kinase (MAPK) and phosphoinositide 3-kinase (PI3K)/Akt pathways, facilitating the activation of transcription factors required for the expression of pro-inflammatory cytokines and cell cycle regulators, for cell migration, proliferation, and survival [13, 16–19]. Recently, MIF has also been linked to several fibrotic diseases, including liver [20, 21], kidney [22, 23], lung, [24, 25], and bladder [26] fibrosis. Although MIF has both pro-fibrotic [23, 26–28] and anti-fibrotic [22, 29] activities, the precise mechanisms of these functions in fibrosis remain unclear.

By analyzing the gene expression profile between Cavernous Hemangioma (CH), a benign, non-infiltrative, slowly progressive vascular neoplasm of the orbit [30, 31] and BLEL, some authors reported the involvement of T-cell receptor (TCR)-signaling pathway [32], Complement System [33] and B-cell receptor signaling pathways in the pathogenesis of BLEL [34, 35], that confirmed the role played by the inflammatory process in the pathogenesis of BLEL. Here, we sought to identify the mediators of the inflammatory response and highlight their implication in the pathogenesis of the disease.

In initial series of inflammatory cytokine expression profiling, we identified MIF as the main pro-inflammatory cytokines in BLEL and its receptors were also found to be overexpressed in BLEL tissues. In addition, the MAPK and PI3K/Akt pathways, which are downstream of MIF-induced signaling cascades, were activated in BLEL. Furthermore, MIF was significantly expressed in the fibrotic area of BLEL tissues. Therefore, we hypothesized and provided evidence that MIF plays a key role in the pathogenesis of BLEL.

## Methods

### Biological materials

#### Plasma sampling

Blood was collected in EDTA tubes and centrifuged within 2 h following collection at 2000 rpm for 10 min. Then the plasma was transferred into sterile tubes and stored at − 80 °C until subsequent analysis.

#### Tissue sampling

Orbital tissue biopsy from patients with CH and BLEL were collected immediately after surgery. A portion of the collected tissues was stored in liquid nitrogen until subsequent assays. The rest of the collected tissues were fixed in formalin, embedded in paraffin, cut into thin slices on a microtome, and mounted onto slides for downstream histological analysis.

#### Primary cell isolation

Freshly collected tissue biopsies were washed with 1x phosphate-buffered saline (PBS) to release blood, incubated with penicillin-streptomycin for at least 1 min, and washed again with PBS. Then the washed tissues were minced into small pieces (approximately 5 μm in diameter) in a sterile 10 cm petri dish, and the washing steps were repeated as mentioned above. The minced tissues were seeded in a Corning® T25 culture flask with a filter cap for adherent cells, incubated for several minutes, and cultured at 37 °C in the presence of 5% CO_2_ with full medium as follows: DMEM/High glucose (HyClone®; Thermo Fisher Scientific, Waltham, MA, USA) supplemented with 10% fetal bovine serum (Gibco, Gaithersburg, MA, USA) and 1% of 10,000 units/mL penicillin and 10,000 μg/mL Streptomycin (Gibco). The medium was changed twice a week. Cells were passaged when they reached 80–90% confluence, and cells from passages 4–12 were used for subsequent experiments.

### Hematoxylin and eosin and Masson trichrome staining

*Hematoxylin and eosin (H&E) staining* was performed as previously described in Cold Spring Harbor Protocols by Fischer A.H. et al. [36] and Masson trichrome staining performed with Masson staining kit (Heart Biological Technology, Xian, China), in strict accordance with the manufacture protocol.

### Microarray analysis

Orbital CH and BLEL tissue biopsies microarray data deposited in gene expression omnibus by Jianmin Ma et al. under the accession number GSE76497 were used for genes expression profiling and for functional annotation. Background correction, quantile normalization and summarization of the expression data were performed under GeneSpring version 14.9 (Agilent Technologies). The significant DEGs were selected with a false discovery rate (FDR) < 0.01 and │FC ≥ 2│and functional annotations performed with PANTHER online tool [37] and Cytoscape plug-in ClueGO version 2.5.

### Cytokines profiling

#### Bio-Plex cytokine assay

Cytokines profiling in plasma and tissue lysates were carried out respectively with Bio-Plex Pro™ Human Inflammation Panel 1, 37-Plex and Bio-Plex Pro™ Human Th17 Cytokine Assays (BIORAD). All the processes were carried out strictly as recommended by the provider. In brief, 96 well pre-wet filter plates were pre-incubated with multiplex bead working solution, washed twice and incubated with standards or samples on a shaker (300 rpm) for 30 min at room temperature. Subsequent to samples and standards incubation, the wells were washed and incubated in dark with detection antibodies (30 min at room temperature, 300 rpm) and then with streptavidin-PE (10 min at room temperature, 300 rpm). After washing, beads in each well were resuspended with Bio-Plex assay buffer and plates read on the Bio-Plex system.

#### Enzyme-linked immunosorbent assay

Plasma collected from both healthy and BLEL patients were analyzed using RayBio human MIF ELISA kit (RayBiotech.Inc). After the recommended incubation time for standards and plasma in wells coated with antibody specific for human MIF, the wells were washed and a biotinylated anti-human MIF antibody is added. Subsequent to 1 h incubation, unbound biotinylated antibodies were washed out and an HRP-conjugate streptavidin solution was added to the wells and washed at the end of the incubation time. The reactions were stopped after incubation with the TMB substrate reagent and optical density read at 450 nm. A standard curve was used to determine MIF concentration for each sample.

### Proliferation and apoptosis assays

#### Cell counting Kit-8 assay

For the proliferation assay, cells were seeded from the same cell suspension at a density of 2 × 10^3^ cells per well in 96-well plates, and incubated for 24 h in full medium only, full medium containing hrMIF (5–400 ng/mL, SRP3321; Sigma Aldrich, St. Louis, MO, USA), or full medium supplemented with the MIF inhibitor ISO-1 (100 μM). Cell proliferation was determined after 24 h and 48 h using the CCK-8 assay.

#### TUNEL assay

Apoptosis analysis in cell cultures and paraffin-embedded tissue sections were performed using the one-step TUNEL apoptosis assay kit in accordance with the manufacturer’s protocol (Beyotime Biotechnology, Shanghai, China). In brief, for detection in tissues, paraffin sections were first dewaxed and rehydrated as we previously reported [38]. For detection in cells, cells were washed, fixed in 4% paraformaldehyde, and permeabilized with 0.3% Triton X-100 in PBS. The labeling reactions were performed for 1 h at 37 °C with 50 μl TUNEL reagent, washed with PBS, and then incubated with a streptavidin-horseradish peroxidase conjugate for 30 min at 37 °C and developed using DAB for 10 min or more if needed. DNase I-treated cells labeled or unlabeled with TUNEL reagents were used as positive and negative, controls respectively. For the TUNEL assay, the cells were seeded in a 24-well plate at a density of 2 × 10^4^ cells per well, incubated for 24 h, and cultured daily for 1 week in full medium only, full medium containing hrMIF at 100 ng/mL or 200 ng/mL or full medium supplemented with 100 μM ISO-1 (Ab142140; Abcam, Cambridge, UK). In the apoptosis induction assay, cells were pretreated with or without 200 ng/mL hrMIF for 48 h, followed by treatment with 1% DMSO (78.13 g/mol; Sigma Aldrich) for 24 h in the presence or absence of hrMIF (200 ng/mL).

#### Annexin V-PE flow cytometry

Apoptotic cell detection by flow cytometry was performed with the Annexin V-PE/7-AAD Apoptosis Kit (MultiSciences Biotech Co, Ltd., Beijing, China). Cells were cultured daily for 1 week in full medium only, full medium containing 200 ng/mL hrMIF (SRP3321; Sigma), or full medium supplemented with 100 μM ISO-1 (Ab142140; Abcam). In accordance with the manufacturer’s protocol, cells were collected and washed twice with PBS, after which the cell pellets were resuspended in 100 μL binding buffer (1x) to a final density of 10^5^–10^6^ cells/reaction tube. A total of 5 μL Annexin V-PE and 10 μL 7-AAD were added to each tube and incubated for 15 min in the dark at room temperature. The analysis was performed on the guava easyCyte 8HT Benchtop Flow Cytometer (Millipore, Stafford, VA, USA) following suspension in 380 μL 1x binding buffer.

### Western blotting

Proteins were extracted from tissues biopsies and from primary cells treated daily for 3 days or 1 week with either rh-MIF (200 ng/ml) or ISO-1 (100 μM) or from untreated cells, using Sangon Biotech tissue and cell proteins extraction kit (Sangon Biotech, Shanghai, China). An equal amount of denatured proteins (in sodium dodecyl sulfate (SDS) at 95 °C for 10 min) was electrophoresed on 12% SDS-PAGE gels, transferred to a nitrocellulose membrane and blocked with 5% BSA blocking buffer at room temperature for 2 h. Subsequent to the blocking step, membranes were incubated overnight at 4 °C with the appropriate first antibody (Additional file 1). After a washing step with 0.01% TBST, membranes were incubated at 37 °C in dark for 1 h with goat anti-mouse or anti-rabbit IRDye® 800C antibody (LI-COR®) and signals were detected and analyzed using LI-COR® Odyssey infrared imaging system.

### Immunohistochemistry and immunofluorescence assays

#### Tissue immunostaining

Paraffin-embedded tissues sections were dewaxed, rehydrated respectively in xylene, ethanol and in distilled water and antigens unmasked in citrate buffer by subsequent heating at 90% for 3 min followed by 40% for 10 min. After the slices cooled at room temperature and washed in distilled water, endogenous peroxidase activity was quenched for 10 min in 3% hydrogen peroxide. For immunohistochemistry assay, sections were thereafter blocked for 2 h at room temperature to prevent unspecific antigen binding and incubated overnight at 4 °C in a humidified chamber with appropriate primary antibody (Additional file 1). Subsequent to an incubation with the first antibody followed by a treatment with a polymer helper, sections were washed and incubated with a Poly-HRP anti-mouse or anti-rabbit IgG antibody (ZSGB-BIO, China), washed and stained with DAB chromogen (ZSGB-BIO, China) and counterstained with hematoxylin. The washed sections were then dehydrated, mounted and viewed under a microscope. For the immunofluorescence assay, the tissues sections were blocked overnight at 4 °C, washed and incubated for 1 h 30 min at 37 °C with appropriate primary antibody (Additional file 1), washed with PBS and incubated in dark at 37 °C for 1 h with the appropriate fluorescent dye-labeled secondary antibodies (Additional file 1). Cell nuclei were stained with NucBlue Live cell stain (R37605, Invitrogen) as recommended by the providers.

#### Cells immunofluorescence staining

*Twenty four well plates were seeded from the same* primary cells suspension in a 24 wells plate at 2.10^4^ cells/well, let been adherent for 24 h and then switched to experimental conditions by addition of a full medium only or full medium containing if needed, rh-MIF (5, 50, 100, or 200 ng/ml) or ISO-1 (100 μM) only or with ISO-1 (100 μM) in presence of rh-MIF. Wells containing the seeded cells were washed with 1X PBS immediately at the end of the incubation times and fixed in 4% paraformaldehyde for 30 min. Subsequent to the fixation, cells were permeabilized when necessary with 0.3% PBS-Tween for 15 min, washed and blocked with normal sheep serum blocking buffer (ZSGB-BIO, China) for at least 1 h at room temperature. Primary antibodies (Additional file 1) have been added at the dilution recommended by the providers and incubated overnight at 4 °C. An appropriate fluorescent dye-labeled secondary antibodies (Additional file 1) was used and cell nuclei stained with NucBlue Live cell stain (R37605, Invitrogen).

Appropriate negative controls: no primary antibody control and labelling control [39] were included in each set to ensure the specificity of the binding of the primary and secondary antibodies to their target and to exclude endogenous fluorescences.

### Statistical analysis

Data were plotted with GraphPad Prism v6.01 and presented as mean ± SEM. Comparison between groups was performed with unpaired t-test, multiple t-tests or one-way analysis of variance with Tukey’s multiple comparisons test. Correlations between MIF and other cytokines levels were assessed by the non-parametric Spearman test using IBM SPSS Statistics 20. Cellprofiler colocalization pipeline (http://cellprofiler.org/examples/#Colocalization) was used on Cellprofiler version 2.2.0 (Cellprofiler™ [40]) to analyze the percentage of Ki-67 positive cells. Test with a *P*-values < 0.05 was considered as significant.

## Results

### Clinical features of BLEL pathogenesis

A total of 99 BLEL patients recruited between December 2011 and April 2016 were included in this study. The gender ratio was 3.26:1 women to men and the median age was 46.5 years (range, 18–80 years). Swelling of lacrimal glands was observed in all patients, with approximately 65.66% (65/99) experiencing eyelid swelling of both eyes, which continued for more than 6 months in most patients (65/95, 68.42%). Tumor size was documented in only 34 of the 99 patients, and in most, the tumor did not reach the organ limit (< 3 cm) (Table 1).

**Table 1:** Summarized clinical information of the BLEL patients

	Gender					
	Female			Male	NA	Total
Gender	75			23	1	99
Median Age (Years)	46 [20–73]			51 [18–80]	–	46.5 [18–80]
Eyelid Swelling						
Both	47			18		65
Left	18			4		22
Right	10			1	1	12
Swelling duration						
≤ 6 months	23			7		30
[7–12 months]	17			8		25
> 12 months	33			6	1	40
NA	2			2		4
		*NA*	*≤6 months*	*[7–12 months]*	*> 12 months*	
Tumor size (cm)	NA	4	20	19	22	65
	< 3 cm	0	8	5	13	26
	3-5 cm	0	2	1	5	8

*NA* Not Available

### Histological and molecular features of BLEL pathogenesis

**Fig. 1:**
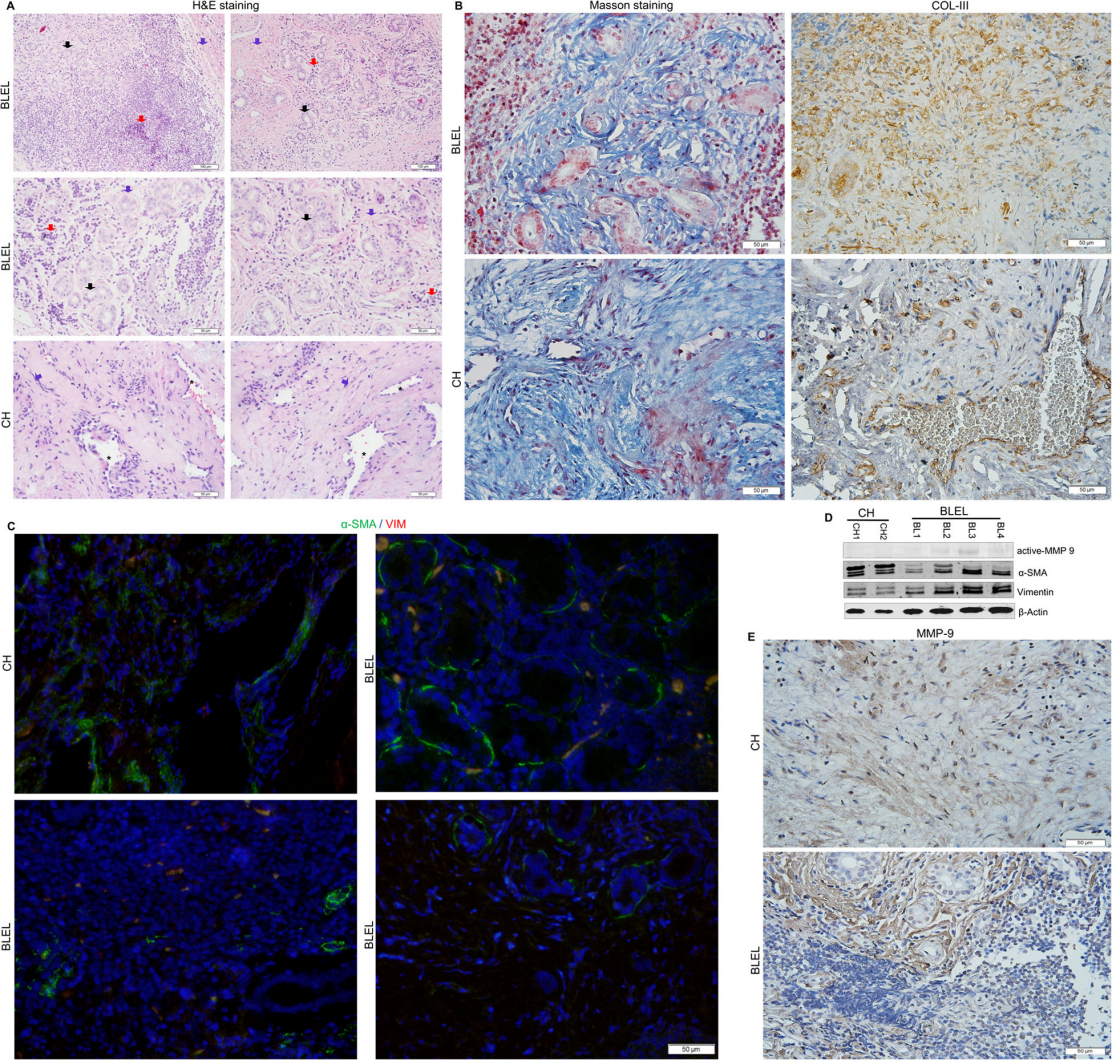
Histopathological features of BLEL. **a** H&E staining showing lymphocytic infiltration in BLEL tissues (red arrows), acinar atrophy (black arrows) and fibrotic area (blue arrows). In the CH panel, * indicates dilated blood vessels while the blue arrows indicated cells with fibroblast-like morphology. **b** The left panel indicates Collagen fibers stained in blue with Masson’s Trichrome. The right panel: immunohistochemistry with a collagen type III antibody detected collagen synthesis and identified the collagen-producing cells in BLEL and CH tissue biopsies. **c** Vimentin-positive fibroblasts (red), α-SMA expressing cells (green) and myofibroblasts (yellow) co-expressing vimentin and α-SMA were observed in BLEL and CH. Myofibroblasts (yellow) were mainly localized in the fibrotic area in BLEL tissues. (**D**) α-SMA and vimentin expression were also confirmed by western blot. The MMP9 expression has also been accessed by western blot (**d**) and immunohistochemistry (**e**). The staining and the immunohistochemistry results presented in this figure are representative of experiments performed on: H&E staining (CH: *n* = 30; BLEL: *n* = 50); Masson staining (CH: *n* = 5; BLEL: *n* = 5); Collagen type III immunostaining (CH: *n* = 4; BLEL: *n* = 4); vimentin and α-SMA co-expression (CH: *n* = 4; BLEL: *n* = 5); MMP9 immunostaining (CH: *n* = 4; BLEL: *n* = 4) tissue biopsies. Original magnification: 20× for images with a 100 μm scale bare or 40× for those with a 50 μm scale bare

Histological analysis of paraffin-embedded tissue sections using hematoxylin and eosin (H&E) staining (Fig. 1a) showed acinar atrophy, massive lymphocyte infiltration, and different degrees of fibrosis in all BLEL specimens, as previously reported [1, 7, 41]. In the control group comprising patients with orbital cavernous hemangioma (CH), a type of blood vessel malformation in which a collection of dilated blood vessels forms a benign tumor, a higher degree of fibrosis and absence of infiltrates were notable (Fig. 1a). Masson trichrome staining revealed excessive deposition of extracellular matrix (ECM) components in the affected tissues with enhanced collagen deposit around epithelial acini in BLEL tissues, thereby confirming fibrosis in BLEL (Fig. 1b). To determine the source of the ECM component deposit, we examined the expression of collagen, one of the main ECM components [42]. In BLEL, collagen type III (Col III) was mainly expressed by acinar epithelial and fibroblast-like cells, whereas in CH, it was mainly expressed by cells surrounding the dilated blood vessels (Fig. 1b). During fibrosis, myofibroblasts (alpha-smooth muscle actin [α-SMA]-expressing cells) serve as the primary collagen-producing cells [43, 44], which raised the possibility that the collagen-expressing cells in both BLEL and CH tissues were of myofibroblast phenotype. Indeed, α-SMA was highly expressed in both BLEL and CH specimens (Fig. 1c, d). Its expression was mainly observed in fibroblast-like cells, myoepithelial cells surrounding the acinar epithelial cells in the BLEL group, and around cells surrounding the dilated blood vessels in the CH group (Fig. 1c). The α-SMA expression profile was consistent with collagen expression and deposit (Fig. 1b, c) in the affected tissues. The ECM is a highly dynamic structure that continuously undergoes controlled remodeling, a process that is mediated by specific enzymes including various families of proteases such as metalloproteinases (MMPs) [45, 46]. Thus, in addition to the excessive ECM deposit observed in BLEL and CH tissue biopsies, MMP-2 and MMP-9 expression was analyzed. MMP-2 was undetectable in both CH and BLEL specimens (data not showed), and only a mild expression of MMP-9 was observed in BLEL tissues (Fig. 1d), including the fibrotic areas and the proximity of acinar epithelial cells (Fig. 1e). Together these data highlighted the occurrence of ECM deposit and confirmed the presence of fibrosis in BLEL.

**Fig. 2:**
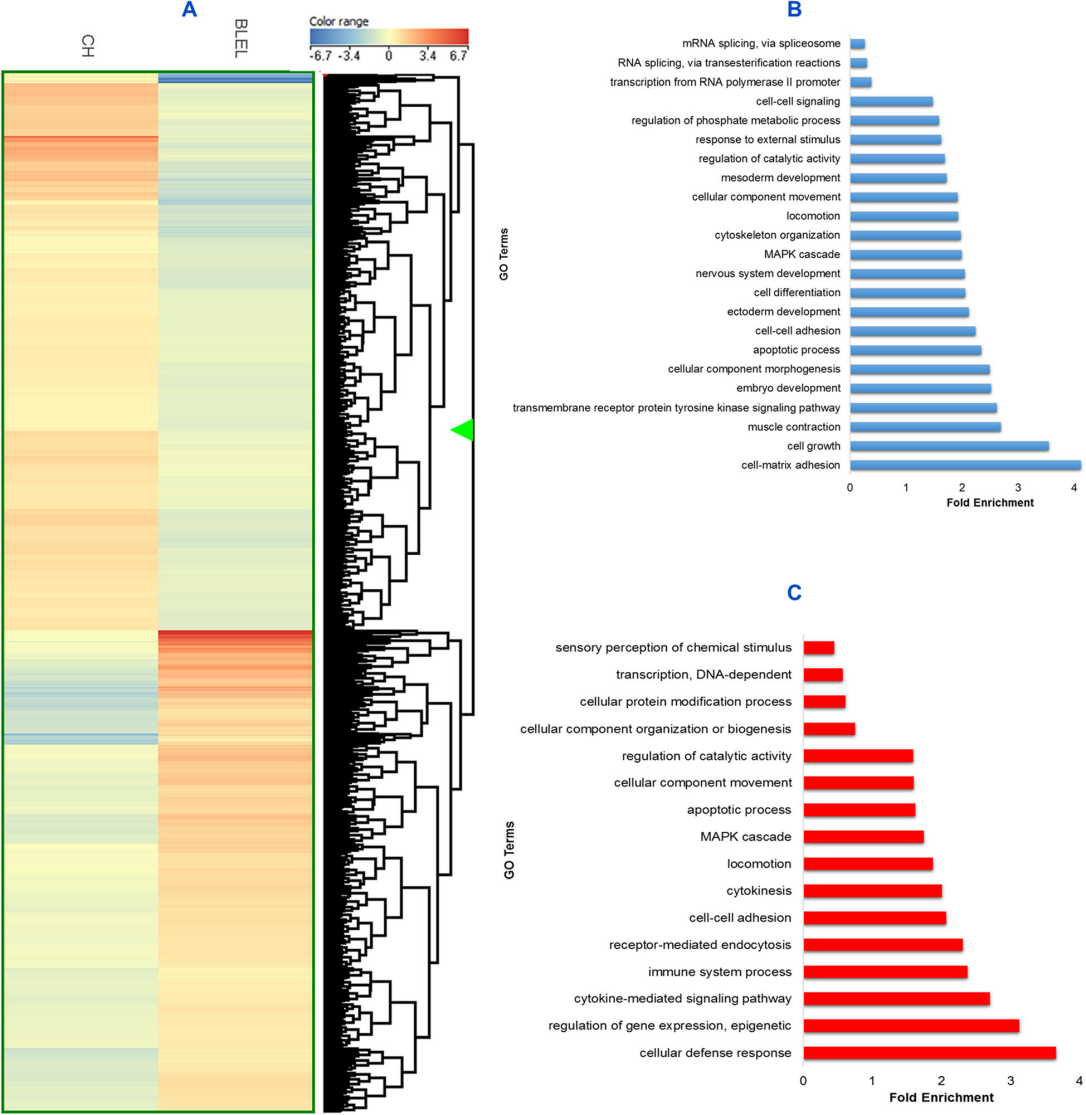
Genes and biological functions associated with BLEL pathogenesis. (**a**) Heatmap of differentially expressed genes (DEGs) in BLEL selected with a FDR < 0.01 and │FC ≥ 2│and biological process related to down-regulated (**b**) and up-regulated (**c**) DEGs are presented. The *GSE76497* dataset was used for the DEGs identification and functional annotation. Sample size: CH *n* = 9 and BLEL *n* = 9, each analyzed in duplicate

To better understand the molecular mechanisms underlying the pathogenesis of BLEL, we performed microarray-based gene expression profiling to identify significant biological functions and pathways. Upon analysis of CH (control group) and BLEL dataset obtained from the Gene Expression Omnibus database (GSE76497), 4409 differentially expressed genes (DEGs) were identified (false discovery rate ≤ 0.01 and │FC ≥ 2│) (Fig. 2a), of which 2006 were upregulated and 2403 were downregulated in BLEL compared with CH (Additional file 2). The upregulated genes were mainly enriched in biological functions and pathways related to immune and inflammatory responses, and the downregulated genes were mostly related to cell adhesion and developmental processes. Enrichment of biological functions such as apoptotic processes, proliferation, the MAPK cascade, and cell adhesion were also observed (Fig. 2b, c, Additional file 3: Figure S1A, B).

### Inflammation-related cytokines profiling identified MIF as the most overexpressed cytokine in BLEL

**Fig. 3:**
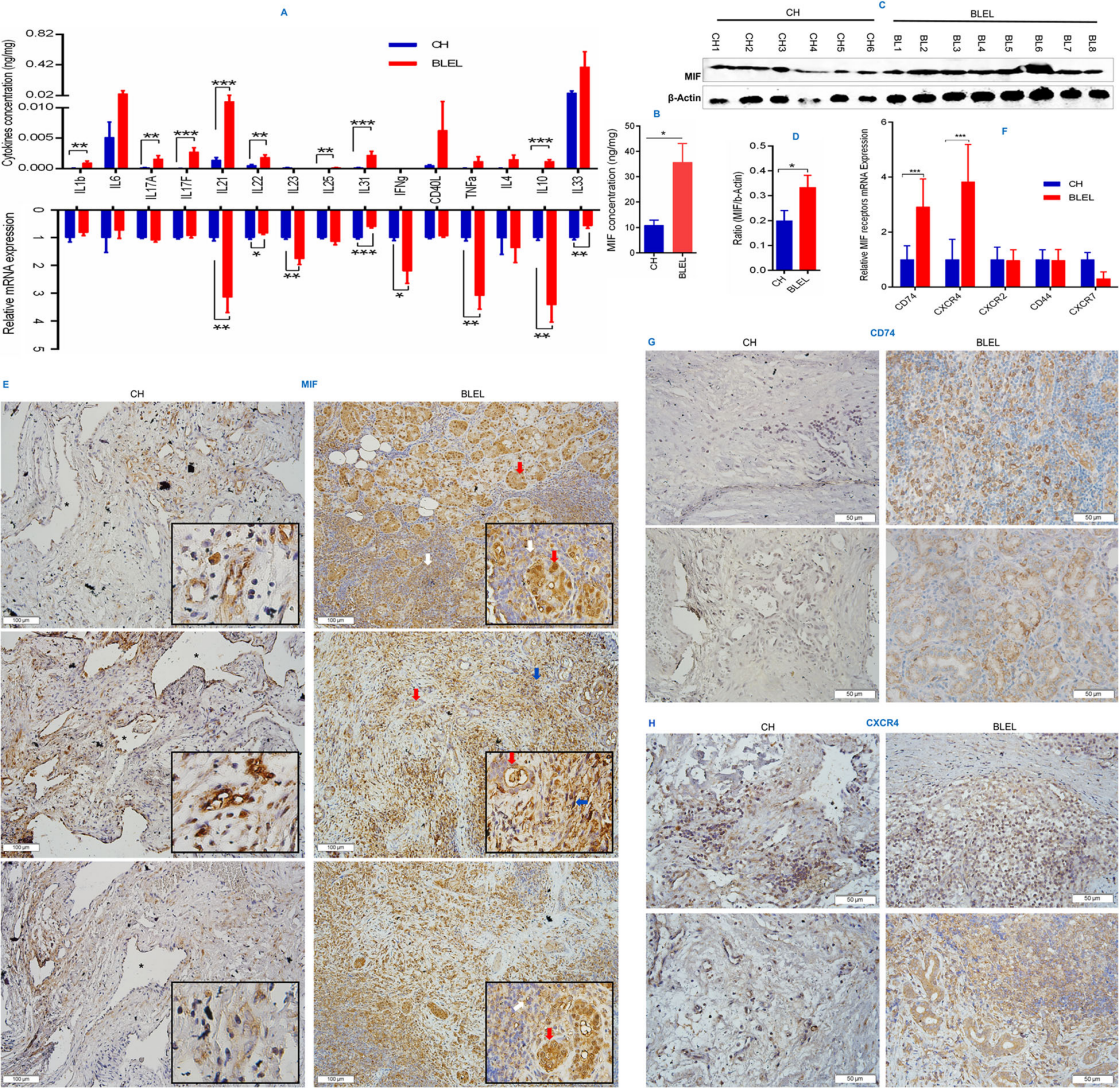
Inflammation-related cytokines expression in BLEL tissues. (**a**) Bio-Plex multiplex cytokine detection in tissue lysates (*n* = 8: 4 CH, 4 BLEL). (**b**) Quantitative analysis of MIF by ELISA in tissue lysates (n = 9: 5 CH, 4 BLEL). (**c**-**e**) MIF expression and distribution in BLEL and CH tissues: (**c** & **d**) are western blot results from tissues obtained from 6 CH and 8 BLEL patients and (**e**) their corresponding immunohistochemistry showing MIF distribution in these tissues. The *Red arrows* indicate expression in acinar cells, the *white arrows* the expression in infiltrated lymphocytes and the *blue arrows* the expression by fibroblast-like cells. (**f**-**h**) highlight MIF receptors expression and distribution in BLEL and CH tissues; (**f**) microarray analysis of MIF receptors mRNA expression (from *GSE76497* dataset); the immunostaining was performed with CD74 (**G**) and CXCR4 (**h**) antibodies on tissues biopsies of 5 CH and 5 BLEL independent specimens. Data in (**a**, **b**, **d** and **f**) were plotted as Mean ± SEM. * *P*-value < 0.05; ** *P*-value < 0.01; *** *P*-value < 0.001; no stars for *P*-value > 0.05; Original magnification: 20× for images with a 100 μm scale bare or 40× for those with a 50 μm scale bare

Although the etiology of BLEL pathogenesis remains unknown, it is thought to be an inflammatory disorder [5, 6, 47]. Our gene expression profiling results from BLEL tissues identified a set of genes associated with immune processes and cytokine secretion. We analyzed inflammation-related cytokines in the plasma of BLEL patients compared to healthy donors using a panel of 38 cytokines from different cytokine families including the pro-inflammatory cytokine MIF, interleukins (ILs), tumor necrosis factor (TNF) superfamily, interferons (IFNs), and MMPs. A significant increase was only observed for MIF and IL-32, whereas IL-8, sIL6-Rβ (gp130), sTNF-R1, MMP2, and MMP3 were downregulated in the plasma of BLEL patients (Additional file 3: Figure S1C). MIF was 22.55-fold higher in BLEL patients whereas only a 4.15-fold increase was observed for IL-32, making MIF the most upregulated of the detected cytokines in the plasma of BLEL patients. Although only MIF and IL-32 were significantly increased in the plasma of BLEL patients, the upregulated DEGs were enriched in pathways involving Th1, Th2, and Th17 cells (Additional file 3: Figure S1B). Moreover, enhanced mRNA expression was observed for the Th1-related cytokines IFN-γ, IL-12A, and TNF; the Th17-related cytokines IL-21 and IL-23A; the Th2-related cytokine IL-10; and other inflammatory cytokines including IL-1A, IL-16, IL-17C, and IL-32 (Fig. 3a, Additional file 3: Figure S1D), suggesting that the inflammation process in BLEL is mainly localized in the affected tissues and may rely on Th cell-induced immunity. We further examined the cytokine profile in the tissues of these same BLEL patients using a panel of MIF, Th1, Th2, and Th17 cytokines. Enhanced expression of MIF, IL-31, Th17-related cytokines (IL-17A, IL-17F, IL-21, IL-1β, and IL-22), and Th2-related cytokines (IL-25, IL-4, and IL-10) were observed (Fig. 3a, b). No significant expression of the Th1-related cytokines TNF-α and IFN-γ were observed (Fig. 3a). As in the plasma, MIF was also overexpressed in BLEL tissues compared with CH tissues (control) (Fig. 3b). Moreover, a strong positive correlation was observed between MIF and 14 of 15 of the analyzed cytokines (Table 2).

**Table 2:** Correlation Analysis between MIF and other inflammatory cytokines in BLEL tissues

Spearman correlation test	MIF		MIF	IL1β	IL6	IL17A
		Spearman rho	1.00	0.40	−0.40	0.80
		*P*-Value (two-tailed)		0.60	0.60	0.20
	MIF		IL17F	IL21	IL22	IL23A
		Spearman rho	1.000^a^	0.40	0.40	b
		*P*-Value (two-tailed)		0.60	0.60	
	MIF		IL25	IL31	IFNγ	CD40L
		Spearman rho	1.000^a^	0.80	b	0.80
		*P*-Value (two-tailed)		0.20		0.20
	MIF		TNF-α	IL4	IL10	IL33
		Spearman rho	0.80	0.40	1.000^a^	0.20
		*P*-Value (two-tailed)	0.20	0.60		0.80

^a^Significant correlation at 0.01; b: undetectable

### Tissue distribution of MIF and its receptors in BLEL

Given that MIF expression was enhanced in both the plasma and tissues of BLEL patients (Additional file 3: Figure S1C, Fig. 3b), we further confirmed its expression (Fig. 3c, d) and analyzed its distribution in BLEL tissues (Fig. 3e). In BLEL tissues, MIF was mainly expressed by lacrimal gland acinar epithelial cells, infiltrated lymphocytes, and fibroblast like-cells, with strong positive staining observed in the cytoplasm and nuclei of epithelial and fibroblast-like cells, whereas in lymphocyte infiltrates, MIF expression was mainly observed in the cytoplasm. In contrast, in the control group, MIF was mainly expressed by cells surrounding the dilated blood vessels, with its expression mainly localized in the nuclei (Fig. 3e). The biological and cellular activities of MIF primarily rely on interaction with its receptors CD74, CD44, CXCR4, CXCR2, or CXCR7 [16]. Thus, we analyzed their expression and distribution in BLEL and CH tissues (Fig. 3f–h). CD74 and CXCR4 mRNA was significantly upregulated, whereas CXCR7 was downregulated in BLEL tissue compared to CH tissue. No significant difference was observed for CD44 and CXCR2 expression (Fig. 3f)*.* The expression of CD74 (Fig. 3g) and CXCR4 (Fig. 3h) proteins was also observed in BLEL and CH tissues. Both CXCR4 and CD74 had a distribution profile similar to that of MIF in BLEL tissues.

### MIF downstream pathways and related functions are activated in BLEL

**Fig. 4:**
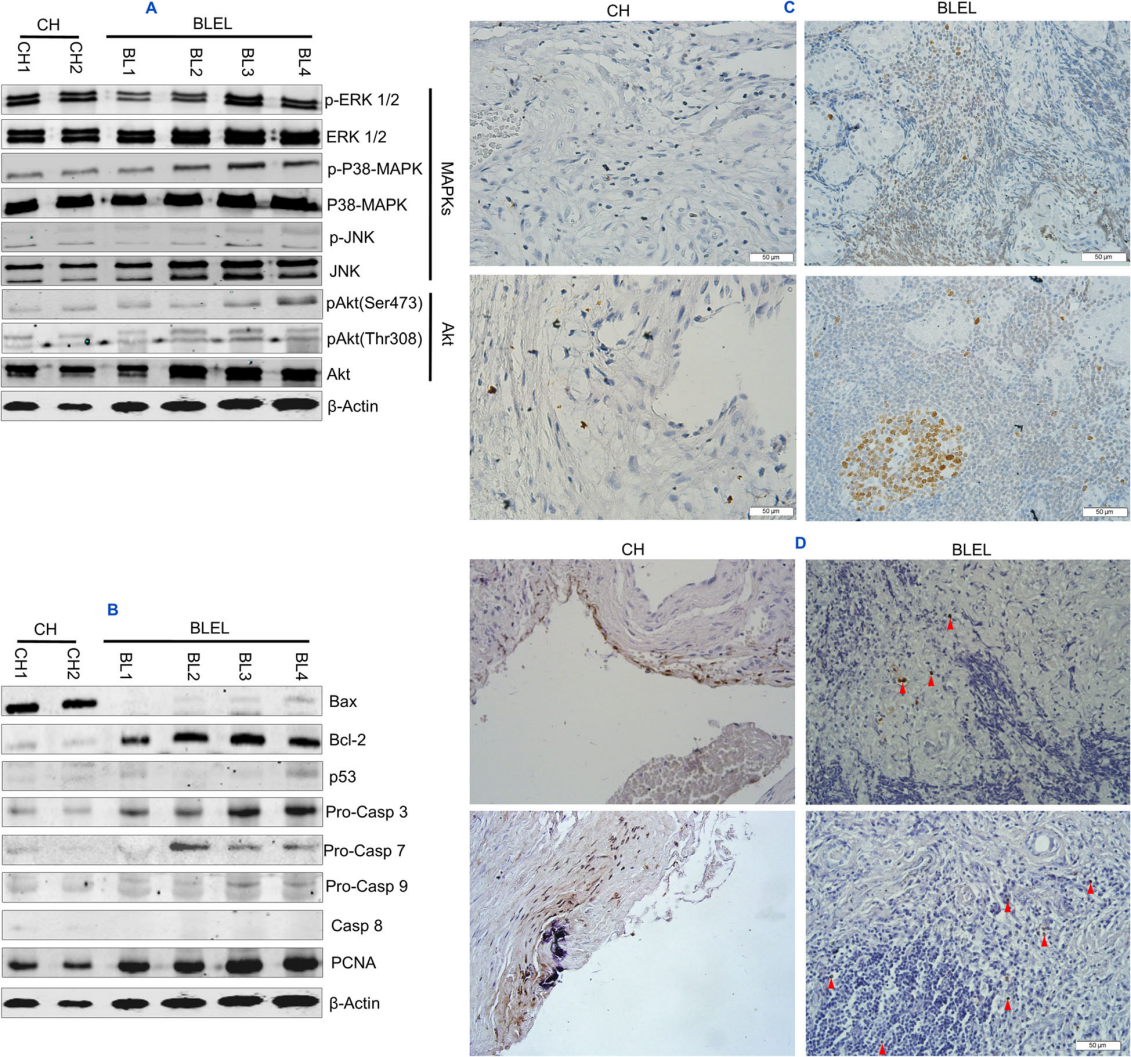
Activation of MAPKs and PI3K/Akt pathways and increased proliferation, and resistance to apoptosis occurred in BLEL. (**a**) Representative western blot results of MAPKs and PI3K/Akt phosphorylation status are shown. (**b**) Representative western blot results of apoptosis and proliferation markers are shown. (**c**) Immunostaining performed for the proliferation marker Ki-67 are shown (CH: *n* = 4; BLEL: *n* = 4). (**d**) Representative images of TUNEL DNA fragmentation assay performed on tissue biopsies of 5 CH and 5 BLEL specimens. Arrowheads in BLEL panel indicated the only few apoptotic cells. (**c** and **d**) Original magnification: 40×

MAPK and PI3K/Akt are the main downstream of MIF-induced signalization [16]. The gene expression profiling in BLEL identified a set of DEGs that were enriched in MAPK and PI3K/Akt cascades. Moreover, phosphorylated MAPK family members extracellular signal-regulated kinase 1/2 (ERK1/2) at Thr202/Tyr204, p38-MAPK at Thr180/Tyr182, and stress-activated protein kinase/c-Jun N-terminal kinase (SAPK/JNK) at Thr183/Tyr185 were detected in BLEL, as well as partial and fully activated Akt at Thr308 and Ser473 respectively, confirming the roles of MAPK and PI3K/AKT pathways in the pathogenesis of BLEL (Fig. 4a).

**Fig. 5:**
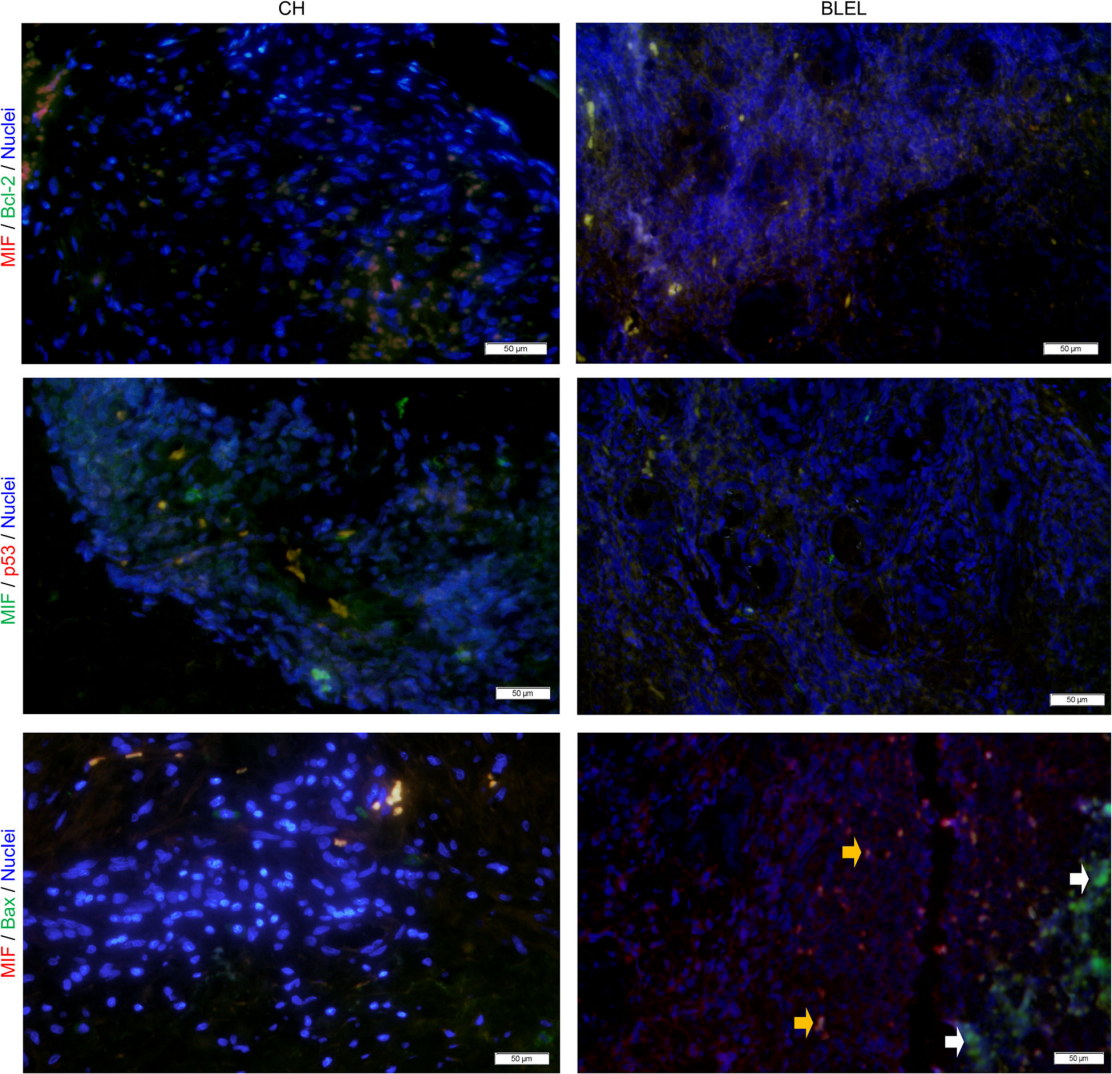
MIF and apoptosis factors expression in BLEL tissues. Double-label fluorescent immunohistochemistry evaluating the co-expression between MIF and Bcl-2, p53 and Bax proteins in BLEL tissues. Representative images of fluorescent immunostaining performed on tissue biopsies of 4 CH and 4 BLEL specimens were shown. Original magnification: 40×

Activation of MAPK and PI3K/Akt pathways regulate inflammation and tissue homeostasis by controlling cell proliferation, differentiation, survival, and migration [48–51]. In BLEL, we observed an increased expression of the proliferating cell nuclear antigen (PCNA) and the proliferation marker Ki-67 (Fig. 4b, c), which together with the enrichment of the upregulated DEGs in biological functions related to cell proliferation, demonstrate an enhanced cell proliferation in BLEL. The distribution of Ki-67 in BLEL tissues revealed proliferation of infiltrates, gland epithelial, and fibroblast-like cells with the strongest staining signals observed in infiltrates and fibroblast-like cells (Fig. 4c). From the gene expression profiling data, we observed a set of DEGs enriched in biological functions related to apoptotic processes. Moreover, TUNEL signals were very low in BLEL (Fig. 4d). As shown in Fig. 4b, there was low expression of the apoptosis inducer Bax in BLEL tissue, whereas anti-apoptotic protein B-cell lymphoma 2 (Bcl-2) was highly expressed. The tumor suppressor protein p53, which induces growth arrest and apoptosis, was not highly expressed in BLEL tissues and had cytoplasmic localization (Fig. 4b, Additional file 3: Figure S1E). Apoptosis is mainly mediated by caspases, which cause cell death through their protease activities [52]. Therefore, we analyzed the expression of initiator caspases 8 and 9 and effector caspases 3 and 7. Despite the high expression of the proforms of these caspases, their active forms were undetectable (Fig. 4b). Overall, these results showed a low frequency of apoptosis in BLEL. MIF upregulates Bcl-2 expression [53, 54] or inhibits apoptosis by physically interacting with p53, thereby preventing its translocation from the cytoplasm to the nucleus [55]. In BLEL, MIF was coexpressed with Bcl-2 and colocalized with p53 in the cytoplasm (Fig. 5). Moreover, high MIF-expressing cells showed low or no expression of Bax, whereas high Bax-expressing cells had little MIF staining (Fig. 5). These data suggest that MIF may be associated with the resistance to apoptosis observed in BLEL.

### MIF regulates biological functions and pathways involved in BLEL pathogenesis

To directly link MIF to the pathologic features observed in BLEL, we evaluated its effects on cell proliferation, apoptosis, and the MAPK and PI3K/Akt cascades, given that they were shown to play a role in BLEL pathogenesis by our upstream analysis. Primary cells (BLELp1 and BLELp2) established from two different BLEL specimens were used for this purpose. These primary cells have a spindle-like shape with an oval flat nucleus, characteristic of fibroblasts [56], and expressed a high level of MIF and its receptors similar to BLEL tissues (Additional file 4: Figure S2A–C).

**Fig. 6:**
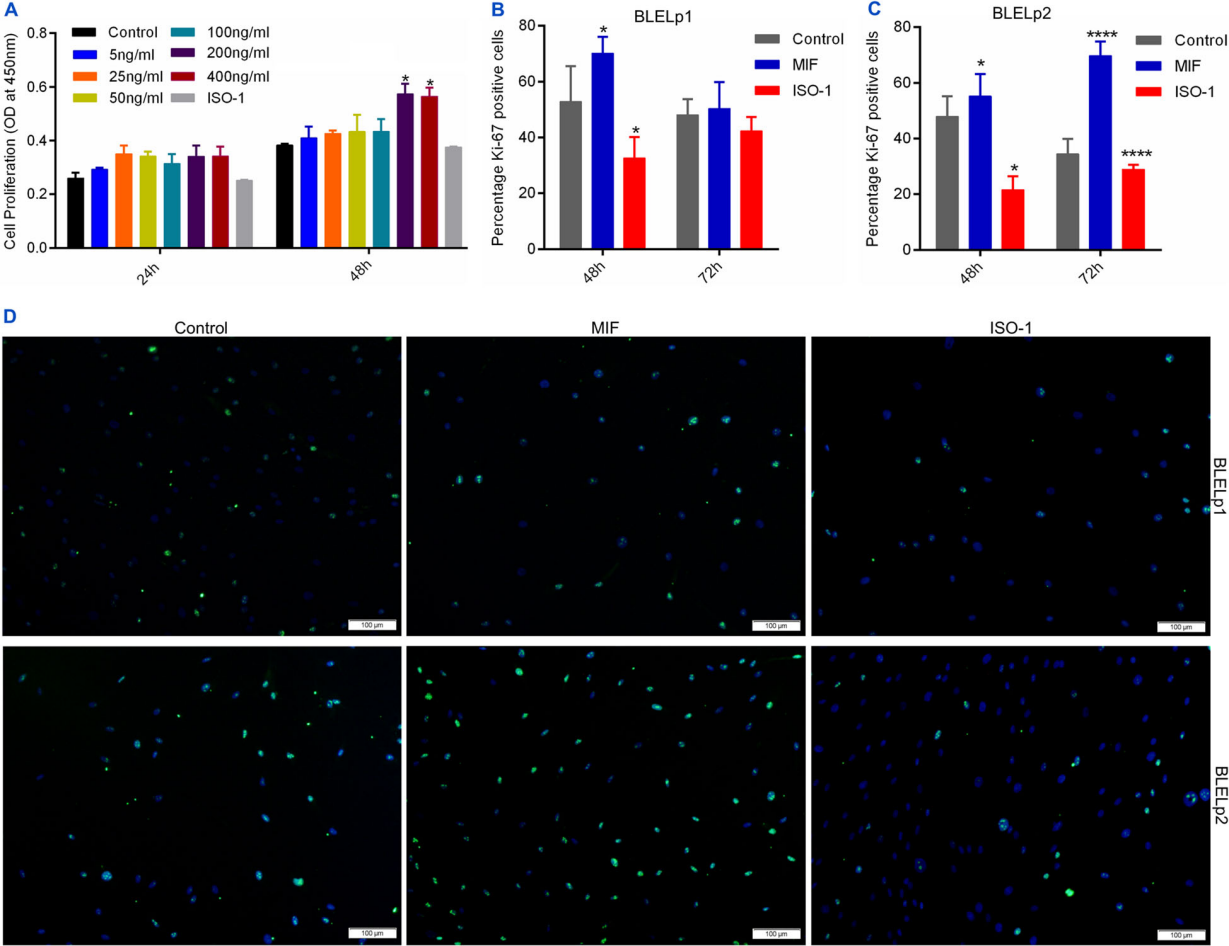
MIF induces proliferation of BLEL-derived primary cells. (**a**) MIF increases BLEL primary cells proliferation in a time and dose-dependent manner. Cells from the same suspension were seeded at a density of 2 × 10^3^ cells per well in 96 well plates, let be adherent for 24 h and cultured with MIF (5 to 400 ng/ml), ISO-1 (100 μM) or without MIF nor ISO-1 at the indicated time point. Viable cells were counted using Cell Counting Kit-8 (CCK8). (**b**-**d**) MIF enhanced the expression of the proliferation marker Ki-67 in BLEL primary cells. Cells from the same suspension were seeded at a density of 2 × 10^4^ cells/well in a 24 wells plate, let be adherent for 24 h and cultured with MIF (200 ng/ml), ISO-1 (100 μM) or without MIF nor ISO-1 at the indicated time point: the percentage of Ki-67 positive cells counted using CellProfiler v2.2.0 (Cellprofiler™) were summarized for (**b**) BLELp1 cells (fibroblast) and (**c**) BLELp2 cells (myofibroblast); (**d**) representative images of the most significant time point were presented for Ki-67 immunostaining: BLELp1 (48 h) and BLELp2 (72 h). Representative results of three independent experiments were presented. Data were plotted as Mean ± SEM. * *P*-value < 0.05; ** *P*-value < 0.01; *** *P*-value < 0.001; no stars for P-value > 0.05. (**d**) Original magnification: 10×

To characterize the effects of MIF on proliferation in BLEL, BLELp1 and BLELp2 cells were exposed to different doses (5–400 ng/mL) of hrMIF, and their proliferation rates were evaluated after 24 and 48 h. Significantly increased proliferation was observed at 48 h, with the most enhanced proliferation observed with treatment of 200 and 400 ng/mL hrMIF (Fig. 6a). Then we examined the effects of MIF (200 ng/mL) on the expression of proliferation markers such as Ki-67 (Fig. 6b–d, Additional file 4: Figure S2D) and PCNA (Additional file 4: Figure S2C), and found that the percentage of Ki-67-expressing cells was significantly enhanced in MIF-treated cells. Although no statistically significant difference was observed in PCNA expression between MIF-treated and untreated cells, its expression increased following prolonged exposure to MIF (1.15-fold increase on day 3 and 1.4-fold increase on day 7). Moreover, treatment with the MIF inhibitor ISO-1 (100 μM) attenuated proliferation and decreased the proportion of Ki-67-positive cells as well as PCNA expression in these cells (Fig. 6a–d, Additional file 4: Figure S2C, D). These data, together with the observed reduced PCNA expression following ISO-1 treatment of lympBLELp2 cells (lymphocytes derived from the same tissue biopsy utilized to culture the BLELp2 cells) (Additional file 5: Figure S3), confirmed the role of MIF in BLEL cell proliferation.

**Fig. 7:**
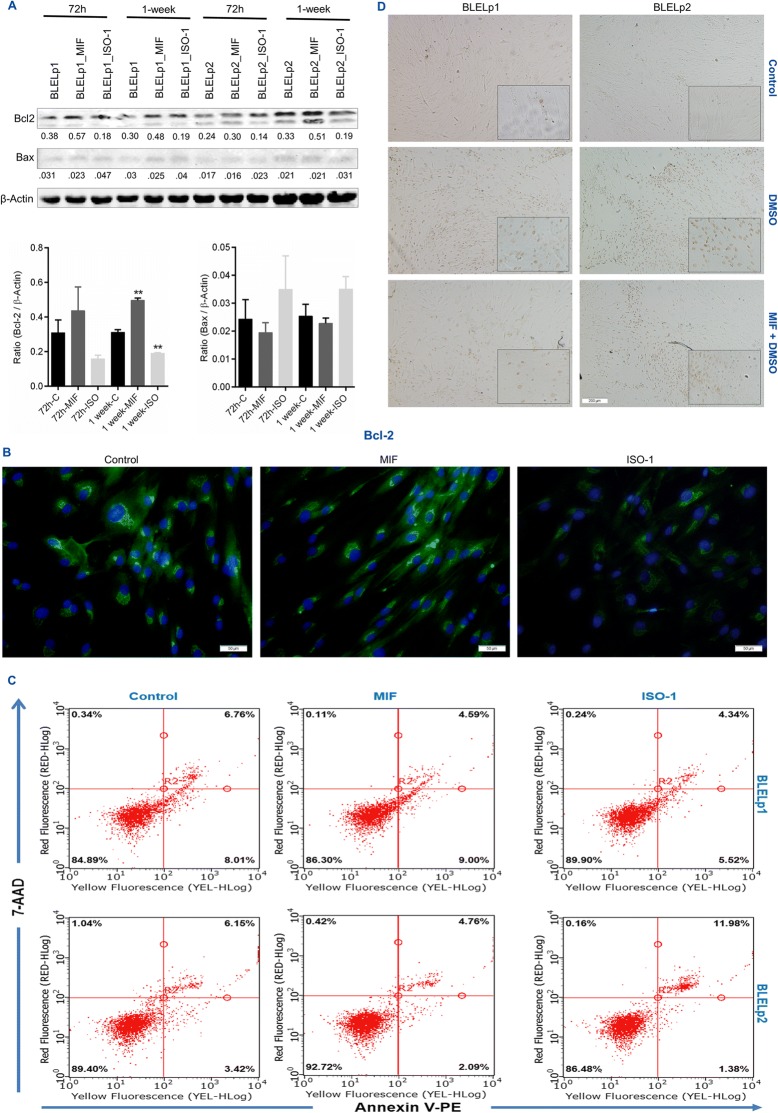
MIF contributes to resistance to apoptosis in BLEL primary cells. (**a**) Immunoblotting showing the influence of MIF on Bax and Bcl-2 proteins expression. (**b**) Representative images of immunostaining showing the influence of MIF on Bcl-2 expression in BLEL primary cells. (**c**) Flow Cytometry graphs showing viable cells (lower-left quadrant), early apoptotic cells (lower- right quadrant), late apoptotic cells (upper-right quadrant) and nuclear debris (upper-left quadrant). (**d**) MIF prevents BLEL primary cells from DMSO-induced apoptosis. In (**a** & **c**) BLELp1 and BLELp2 cells were seeded at a density of 10^6^ cells/dish, and at a density of 2 × 10^4^ cells/well in 24 well plate in (**b** & **d**). Cells were treated with MIF (200 ng/ml), ISO-1 (100 μM) or without MIF nor ISO-1 for 72 h or 1 week in (**a**), and for 1 week in (**b** & **c**). In (**d**), cells were pre-treated with or without MIF (200 ng/ml) for 48 h before exposition to 1% DMSO (78.13 g/mol) for 24 h together with or without MIF (200 ng/ml). Data were plotted as Mean ± SEM. * *P*-value < 0.05; ** *P*-value < 0.01; *** *P*-value < 0.001; no stars for *P*-value > 0.05. Original magnification: (**b**) 20× and (**d**) 4×

To investigate whether the resistance to apoptosis observed in BLEL was due to MIF, BLELp1 and BLELp2 cells treated with hrMIF (200 ng/mL) or ISO-1 for 72 h or 1 week were utilized. There was increased expression of Bcl-2 and decreased expression of Bax in cells treated with hrMIF, whereas the opposite results (decreased Bcl-2 expression and increased Bax expression) were observed when MIF activities were blocked by ISO-1 (Fig. 7a, b). Additional analysis of Bcl-2 expression in LympBLELp2 cells also revealed decreased Bcl-2 expression in response to MIF inhibition with ISO-1 (Additional file 5: Figure S3). Analysis of phosphatidylserine (PS) redistribution on the cell surface by flow cytometry showed that treatment of BLELp1 cells with hrMIF reduced the proportion of late apoptotic cells (1.5-fold decrease), whereas in BLELp2 cells, early apoptosis was reduced as well (1.64-fold and 1.3-fold decrease of early and late apoptotic cells, respectively) (Fig. 7c). In contrast to BLELp1 cells, in which treatment with ISO-1 induced resistance to apoptosis (1.5-fold less apoptosis: 1.4-fold decrease of early and 1.6-fold decrease of late apoptotic cells), early apoptotic BLELp2 cells was reduced (2.5-fold decrease), but the proportion of late apoptotic cells almost doubled (1.95-fold increase) following MIF inhibition with ISO-1 (Fig. 7c). The ability of these cells to undergo apoptosis as well as the ability of MIF to prevent apoptosis, as suggested by PS, Bax, and Bcl-2 expression, was further investigated by stimulation with dimethyl sulfoxide (DMSO) in the presence or absence of hrMIF. Treatments with DMSO caused both BLELp1 and BLELp2 cells to undergo apoptosis, as revealed by the DNA fragmentation assay; however, pretreatment with hrMIF delayed apoptosis (Fig. 7d). Because effector caspases 3 and 7 are crucial in the apoptosis process, we used western blotting to analyze their expression on days 3 and 7 following treatment with hrMIF or ISO-1. Surprisingly, the proforms of these caspases were highly expressed, but the active forms were not detected, even in the presence of ISO-1 (Additional file 4: Figure S2E). Together, these data indicate that MIF is involved in the resistance to apoptosis of BLEL cells, and also suggest a caspase-independent process.

**Fig. 8:**
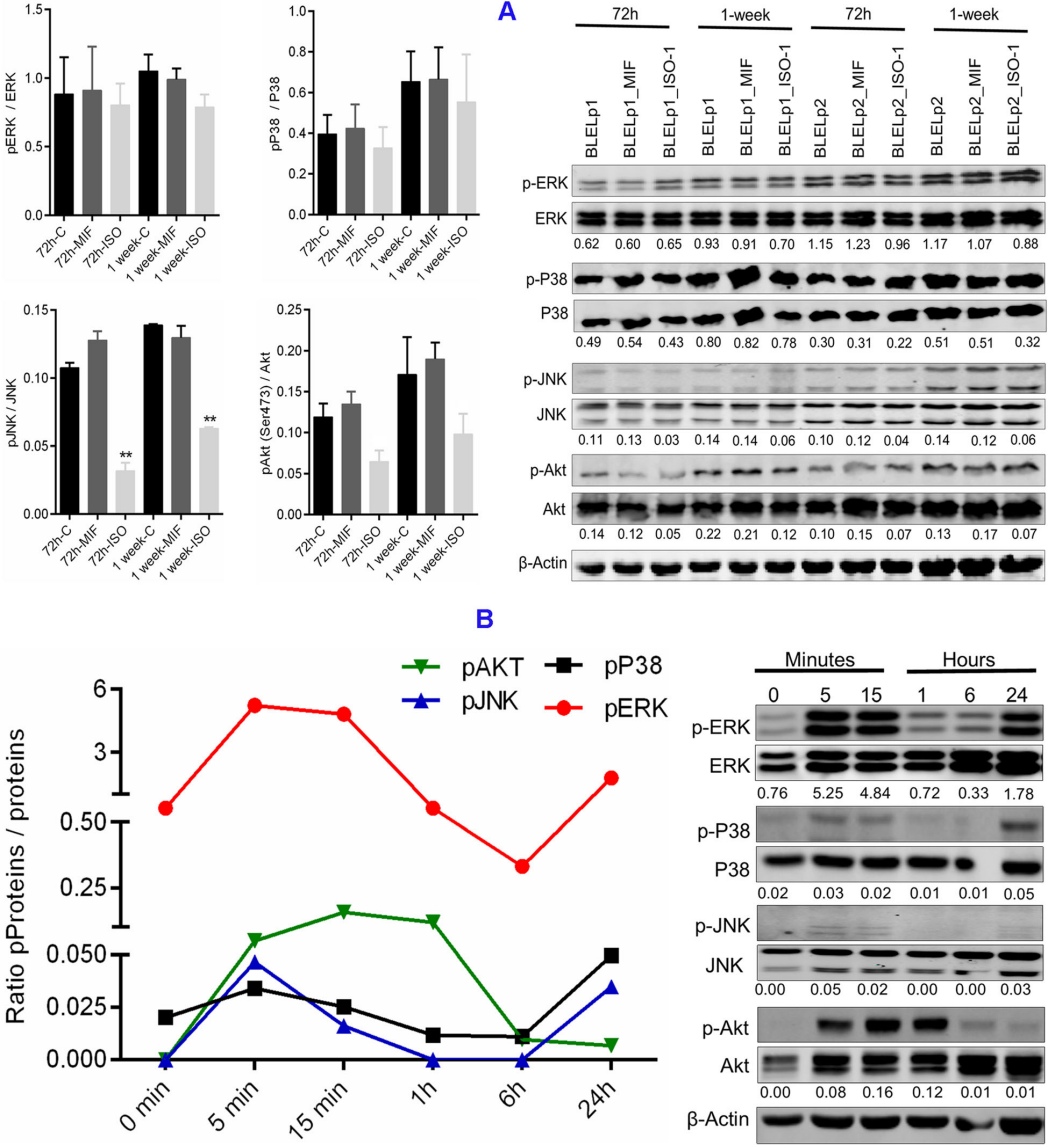
MIF modulates MAPKs and PI3K/Akt phosphorylation in BLEL primary cells. (**a**) Influence of MIF on MAPKs and PI3K/Akt phosphorylation evaluated at 72 h and 1 week following treatment with MIF (200 ng/ml), ISO-1 (100 μM) or without MIF nor ISO-1. (**b**) Immunoblotting showing the kinetic of MIF-induced MAPKs and PI3K/Akt phosphorylation in BLEL primary cells. BLELp2 cells were seeded at a density of 2 × 10^5^ cells/well in 24 well plate, let be adherent for 24 h and stimulated with MIF (200 ng/ml) at the indicated time point. Data were plotted as Mean ± SEM. * *P*-value < 0.05; ** *P*-value < 0.01; *** *P*-value < 0.001; no stars for *P*-value > 0.05

To determine if MIF regulates MAPK and PI3K/Akt pathways, BLELp1 and BLELp2 primary cells were treated for 72 h and 1 week with hrMIF (200 ng/mL) or ISO-1. Treatment with ISO-1 significantly decreased JNK (3.67- and 2.34-fold decrease on days 3 and 7, respectively) and Akt phosphorylation (2.0- and 1.9-fold decrease on days 3 and 7, respectively). However, only a slight decrease in phosphorylation was observed for p38 (1.2-fold decrease on days 3 and 7) and ERK1/2 (1.1- and 1.34-fold decrease on days 3 and 7, respectively) following MIF inhibition (Fig. 8a). In lympBLELp2 cells, increased phosphorylation of ERK1/2 and JNK was observed following MIF inhibition with ISO-1. Moreover active Akt was undetectable (Additional file 5: Figure S3). The absence of a significant change in MAPK and Akt phosphorylation following stimulation with hrMIF (Fig. 8a) in these cells was because their phosphorylation had occurred in the first hour following treatment with hrMIF and was transient (Fig. 8b). These results suggest the differential regulation of MAPK and Akt activities by MIF.

### MIF promotes fibrosis in BLEL

**Fig. 9:**
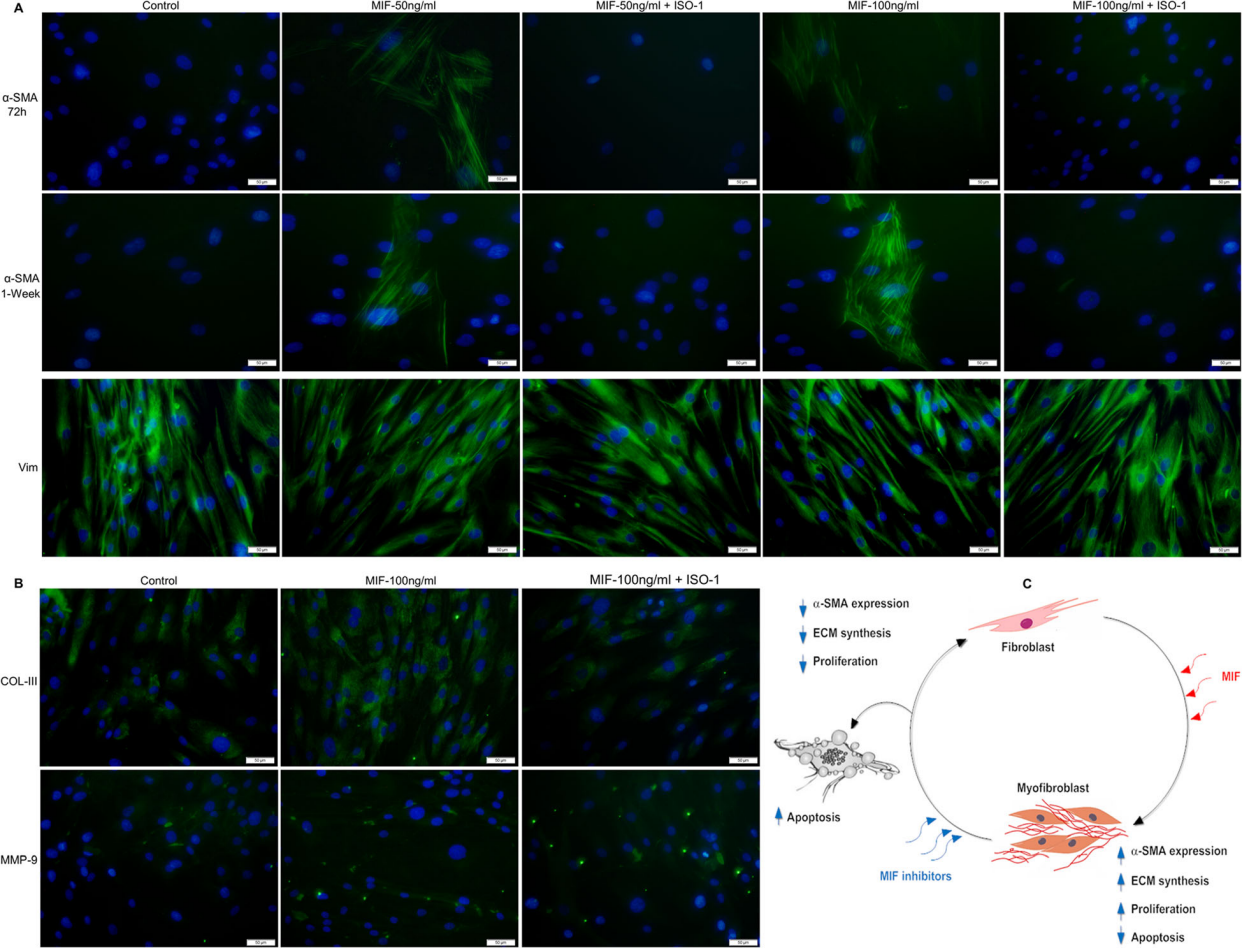
MIF induces myofibroblast phenotype and regulates the synthesis of ECM components in vitro. BLELp1 (fibroblast) cells were treated with MIF or with MIF + ISO-1 at the indicated concentration or without MIF nor ISO-1 for (**a**) 72 h and 1 week, (**b**) for 1 week and analyzed for marker proteins by immunocytochemistry. (**c**) Schematic illustration of MIF functions in fibrotic processes. Original magnification: 20×

Given that in BLEL tissues, increased MIF expression was observed in the fibrotic area (Fig. 3e), and considering previous reports implicating MIF in fibrosis [20, 23, 25–28], we hypothesized that MIF may promote fibrosis in BLEL. To support the concept that fibrosis is typically characterized by fibroblast differentiation into myofibroblasts and excessive ECM synthesis and secretion [43, 44], we determined if MIF could induce the myofibroblast phenotype and regulate ECM synthesis. BLELp1 and BLELp2 cells were used because of their fibroblast-like morphology, which was confirmed by their expression of the fibroblast marker vimentin [57] (Fig. 9a, Additional file 6: Figure S4A). A subsequent investigation revealed that BLELp1 cells expressed vimentin but not α-SMA (control group of Fig. 9a), indicative of a fibroblast phenotype, whereas vimentin and α-SMA were expressed by BLELp2 cells, characteristic of the myofibroblast phenotype (Additional file 6: Figure S4A, B). MIF-containing medium (50 ng/mL and 100 ng/mL) induced α-SMA expression in BLELp1 primary fibroblast cells, and culturing with hrMIF in the presence of ISO-1 blocked the phenotype switch (Fig. 9a). In BLELp2 primary myofibroblast cells, stimulation with 100 ng/mL hrMIF enhanced α-SMA expression, which was significantly downregulated in the presence of ISO-1 (Additional file 6: Figure S4B). Similar results were observed upon stimulation with 200 ng/mL hrMIF (Additional file 6: Figure S4A). However, stimulation with 5 ng/mL hrMIF (data not shown), a MIF concentration that has been found in the plasma of healthy adults [58], did not induce the phenotype switch, suggesting that MIF overexpression is a prerequisite. To assess the ability of MIF to regulate the synthesis of ECM components, its effects on Col III and fibronectin were evaluated in BLELp1 and BLELp2 cells, respectively. Inhibition of MIF downregulated Col III and fibronectin expression, which was enhanced following stimulation with hrMIF (Fig. 9b, Additional file 6: Figure S4B). No significant change, however, was observed in MMP-9 expression among the untreated, MIF-treated, and ISO-1-treated groups (Fig. 9b). Together, these observations suggest that MIF may regulate fibrosis in BLEL.

## Discussion

The present study highlighted several aspects of the pathogenesis of BLEL of the lacrimal gland and showed that MIF is a key player in the progression of this disease. Very little information has been published about the molecular mechanisms underlying the pathogenesis of BLEL, which is assumed to be an inflammatory disease [5, 6, 47, 59]. In the present study, we sought to identify the mediators of the inflammation and provide evidence of their implication in the pathogenesis of BLEL. By comparing the gene expression between CH, a benign tumor which had no clinical or laboratory evidence of systemic autoimmune disease [30, 31] and BLEL, we observed an enrichment of the upregulated DEGs in cellular defense, immune response, and pathways related to infections and autoimmune diseases, which together with the overexpression of toll-like receptor (TLR) mRNA (Additional file 2), suggested that infections and/or autoimmune reactions are involved in BLEL pathogenesis [60–62]. BLEL is an IgG4-related systemic inflammatory disease involving Th2 cell-induced immunity [5, 6, 47, 59]. Inflammatory genes and cytokine profiling in our BLEL group not only implicated Th2 cells in BLEL pathogenesis but also revealed a Th17 cell-related inflammatory response as well as MIF overexpression in this disease. They also suggest that BLEL is a localized, rather than systemic inflammatory disease given that the inflammatory responses were mainly observed in the affected tissue. Indeed, from the 38 cytokines analyzed in the plasma of BLEL specimens, only MIF and IL-32 were significantly overexpressed, whereas in the tissue biopsies of these patients, MIF and IL-31; the Th17-related cytokines IL-17A, IL-17F, IL-21, IL-1β, and IL-22; and the Th2-related cytokines IL-25, IL-4, and IL10 were highly expressed.

MIF is a pleiotropic, pro-inflammatory cytokine known for its role in several inflammatory disorders and tumors [13–15]. MIF cellular activities are initiated through interactions with its proposed receptors CD74, CXCR2, CXCR4, and CXCR7 [13, 16–19]. These interactions result in activation of the MAPK (ERK1/2, p38, and SAP/JNK) and PI3K/Akt pathways, facilitating the activation of transcription factors required for the expression of pro-inflammatory cytokines, cell cycle regulators, and cell migration, proliferation, and survival factors [13, 16–19]. In BLEL specimens, we observed elevated MIF levels in plasma and tissues, which strongly correlated with the expression of most of the analyzed pro-inflammatory cytokines. Moreover, MIF and its receptors were found to have similar tissue distribution profiles that could be favorable to their interaction and their initiation of signal transduction. MAPK and PI3K/Akt signaling leads to numerous fundamental cellular processes, and their dysregulation has been implicated in the development of many human diseases [50, 63–65]. In BLEL, the upregulated DEGs were enriched in genes encoding proteins in the MAPK cascade. Moreover, we noted the phosphorylation of Akt as well as MAPK family members ERK1/2, p38, and SAP/JNK in BLEL tissue biopsies, suggesting their role in BLEL pathogenesis. Considering these observations, in addition to the observed enhanced cell proliferation, the resistance to apoptosis, and leukocyte infiltration in BLEL tissues, it can be hypothesized that MIF has key functions in this pathology.

In vitro, we sought to inhibit MIF by targeting its tautomerase activity with ISO-1 at a concentration of 100 μM, previously reported to be more efficient against the pro-inflammatory activities of this cytokine [66, 67]. In this study, we found that MIF caused the proliferation and survival of BLEL-derived primary cells. Stimulation with MIF accelerated BLEL cell proliferation, while MIF inhibition with ISO-1 significantly delayed proliferation. MIF enhanced the expression of the anti-apoptosis factor Bcl-2, decreased the expression of the pro-apoptotic factor Bax, prevented PS externalization on the surface of BLEL primary cells, which is indicative of resistance to apoptosis [68, 69], and induced resistance to DMSO-mediated apoptosis. Therefore, it appears that MIF act as a proliferation and survival factor in BLEL. Although MIF is linked to resistance to apoptosis in BLEL-derived primary cells, some unexpected results were observed. Indeed, inhibition of MIF with ISO-1 decreased resistance to apoptosis in BLELp2 but not for BLELp1 primary cells. Rather, in BLELp1 cells, MIF inhibition induced resistance to apoptosis. BLELp1 cells are fibroblasts, whereas the majority of BLELp2 cells are myofibroblasts. This phenotype difference explains the contrasting apoptosis results observed following intracellular MIF inhibition; this cell type-specific phenomenon has been previously reported [70–73]. Although MIF inhibition increased the apoptosis of BLELp2 and lympBLELp2 cells, no active caspase 3 or caspase 7 was detected. These results, together with the absence of active caspases in BLEL tissues, demonstrate the MIF contribution to the resistance to apoptosis noted in BLEL and suggest caspase-independent apoptosis signaling in BLEL.

In pathological conditions, several structural and morphological changes that affect the normal functions of the human lacrimal glands can occur [74, 75]. These changes include acinar atrophy, periacinar fibrosis, periductal fibrosis, interlobular ductal dilatation, interlobular ductal proliferation, and lymphocytic and fatty infiltration [75]. In BLEL-tissue biopsies, we noted lymphocytic infiltration and acinar atrophy, together with collagen deposit around acinar and ductal cells, demonstrating periacinar as well as periductal fibrosis in BLEL. Although the exact cause of gland atrophy is unclear, lymphocyte infiltration may be triggered in response to acute or chronic inflammation and/or to infection. Fibrosis must be an end result of inflammation that fails to resolve or of an infection that persists [76, 77]. In BLEL, the pro-inflammatory cytokine MIF was found to be highly expressed in fibrotic areas. Given that MIF has pro-fibrotic functions [20, 23, 26, 28], it was hypothesized that MIF may contribute to fibrosis in BLEL. Considering that fibrosis is primarily mediated by the induction of myofibroblasts, which produce large amounts of ECM components [78], our first question was to determine if MIF could induce the myofibroblast phenotype. Using the BLEL-derived primary fibroblast cells BLELp1 (express vimentin but not α-SMA), we demonstrated that exogenous MIF could induce these BLEL fibroblast cells to differentiate into myofibroblasts; however, this differentiation process required an MIF concentration higher than that observed under normal physiological conditions. Inhibition of endogenous MIF in BLELp2 cells, which are of the myofibroblast phenotype (expressing vimentin and α-SMA), showed a significant decrease in α-SMA expression suggestive of their deactivation [79]. We also showed that stimulation of these BLELp2 myofibroblast cells with MIF considerably enhanced their expression of α-SMA, as well as their proliferation and survival. If we consider that both BLELp1 and BLELp2 cells had high intracellular MIF levels, it seems that extracellular MIF is required for both initiation and maintenance of the myofibroblast phenotype, while intracellular MIF may contribute to maintenance only. Fibrotic disorders are typically associated with excessive ECM deposition [43–46, 76, 78, 80]. Col III and fibronectin are the two main ECM components [42]. In this study, we showed that MIF induced Col III and fibronectin synthesis. Overall, we demonstrated that MIF actively contributes to the fibrosis processes in BLEL primary cells through its ability to induce fibroblast differentiation into myofibroblasts, to enhance myofibroblast proliferation and resistance to apoptosis, thereby ensuring their maintenance, and to stimulate BLEL fibroblasts and myofibroblasts to synthesize ECM components (Fig. 9c). Therefore, it is clear that MIF plays key roles in fibrosis in BLEL pathogenesis.

Our investigation also identified MIF in the regulation of the MAPK and PI3K/Akt pathways in BLEL, as shown either by MIF treatment or MIF inhibition assays. Treatment of BLEL-derived primary cells with MIF induced earlier and transient activation rather than sustained activation of ERK1/2, p38, JNK, and Akt, as evidenced by MAPK/Akt kinetics and the 72-h and 1-week experiments. In BLELp1 and BLELp2 cells, MIF inhibition attenuated ERK1/2, p38, JNK, and Akt phosphorylation. In these cells, JNK and Akt phosphorylation were the most affected by endogenous MIF inhibition, suggesting that endogenous MIF may also be required for activation of JNK and Akt pathways but not for ERK1/2 and p38 pathways. Another interesting finding was the MAPK expression profile post-MIF inhibition in lympBLELp2 cells. In these tissue-derived lymphocytes, increased phosphorylation was observed for ERK1/2 and JNK following MIF inhibition, while these phosphorylation were attenuated in the corresponding myofibroblast cells BLELp2, suggesting that in BLEL, MIF may regulate MAPK and PI3K/Akt signaling differently depending on the cell type. Overall, together with the high level of MIF detected in the plasma of BLEL specimens, these results indicate that both endogenous and exogenous MIF contribute to the regulation of MAPK and PI3K/Akt signaling in BLEL pathology.

Experimental and preclinical studies have shown the efficacy of MIF inhibitors including ISO-1 in autoimmune and inflammatory diseases [81–84]. D-dopachrome tautomerase (D-DT), a homologue of MIF is present in most tissues, circulates at the same concentration and recapitulates all the biological functions of MIF. D-DT and MIF work cooperatively and the neutralization of D-DT or MIF or D-DT and MIF significantly decreases inflammation [85–88]. Here, we provided evidence that MIF is highly expressed in BLEL of the lacrimal gland, which combined with the strong correlation previously revealed between MIF and D-DT concentration [58] suggest that, like MIF, D-DT may also be overexpressed in BLEL and should be in future, considered for further examination in the pathogenesis of BLEL. Recently, Phase I and Phase II studies have been carried out with specific MIF monoclonal antibodies in patients with cancer and systemic lupus erythematosus (SLE) [89–92], and can also be tested in patients with BLEL.

## Conclusions

In the present study, we demonstrated MIF overexpression in BLEL and showed that this pleiotropic cytokine leads to proliferation, induces resistance to apoptosis, and regulates MAPK and PI3K/Akt pathways in BLEL. We also provided evidence that MIF plays a role in fibrosis through its ability to induce fibroblast differentiation into myofibroblasts, enhance proliferation and resistance to apoptosis, and stimulate BLEL fibroblasts and myofibroblasts to synthesize ECM components. Although the direct link between MIF, MAPK and PI3K/Akt pathways, and these MIF-induced functions has not been confirmed in BLEL, based on the already well-established relationship between MAPK, PI3K/Akt, and MIF biological activities, we can infer that MIF may lead to cell proliferation, survival, and fibrosis in BLEL through modulation of the MAPK and PI3K/Akt pathways. We also established a strong positive correlation between MIF and several pro-inflammatory and pro-fibrotic cytokines, suggesting that in BLEL, MIF may also create a microenvironment favorable for inflammation and fibrosis. Moreover, given that MIF counteracts glucocorticoid-induced suppression of cytokines, which are the first-line treatment for most inflammatory diseases [93], MIF emerges as a promising therapeutic target for the treatment of BLEL of the lacrimal gland.

## Additional files

Additional file 1: List of antibodies used in the study. (XLSX 10 kb)

Additional file 2: DEGs, and their GO and KEGG annotations. (XLSX 754 kb)

Additional file 3: **Figure S1.** (**A & B**) KEGG pathways associated to the down-regulated (**A**) and the up-regulated (**B**) DEGs. (**C**) Inflammatory cytokines identified in plasma of BLEL patients. MIF was analyzed from the plasma of 20 BLEL and 25 healthy donors. The other cytokines were assessed from the plasma of 10 BLEL and 4 healthy donors. (**D**) Microarray data showing the relative mRNA expression of the indicated genes. (**E**) Fluorescent immunohistochemistry of p53 expression in BLEL tissues. Representative images of fluorescent immunostaining performed on tissue biopsies of 4 CH and 4 BLEL specimens are shown. (**A & B**) Data were plotted as (*−log10)* of the corrected *p*-value*.* (**C & D**) Data were plotted as Mean ± SEM, unpaired t-test and multiple t-tests with 1% FDR were used. (**E**) Original magnification: 40×. (TIF 1592 kb)

Additional file 4: **Figure S2.** (**A&B**) Showed representative immunostaining results of MIF and its receptors expression in BLEL primary cells. (**C & E**) Immunoblotting showing MIF and PCNA (**C**) and caspases (**E**) expression in different experimental conditions. BLELp1 and BLELp2 cells were seeded at a density of 10^6^ cells/dish and were treated with MIF (200 ng/ml), ISO-1 (100 μM) or without MIF nor ISO-1 for 72 h or 1 week. (**D**) Representative images of Ki-67 immunostaining performed in BLELp1 (72 h) and BLELp2 (48 h). Cells from the same suspension were seeded at a density of 2 × 10^4^ cells/well in a 24 wells plate, let be adherent for 24 h and cultured with MIF (200 ng/ml), ISO-1 (100 μM) or without MIF nor ISO-1 at the indicated time point. Original magnification: (**A and B**) 20× and (**D**) 10×. (TIF 2580 kb)

Additional file 5: **Figure S3.** Immunoblotting showing the influence of MIF on the expression of indicated proteins in BLEL tissue-derived lymphocytes (*LympBLELp2*) following 72 h treatment with MIF (200 ng/ml), ISO-1 (100 μM) or without MIF nor ISO-1. (TIF 568 kb)

Additional file 6: **Figure S4.** Influence of MIF on αSMA and fibronectin expression. (**A**) BLELp1 (fibroblast) and BLELp2 (myofibroblast) cells were treated with MIF (200 ng/ml) or ISO-1 (100 μM) or without MIF nor ISO-1 for 72 h and 1 week, (**B**) BLELp2 (myofibroblast) cells were treated with MIF (200 ng/ml) or ISO-1 (100 μM) or without MIF nor ISO-1 for 1 week. (**B**) Original magnification: 10×. (TIF 6147 kb)

## Abbreviations

BLEL: Benign lymphoepithelial lesion
BLELp: Benign lymphoepithelial lesion primary cell
CD74: Cluster of differentiation 74
CH: Cavernous Hemangioma
CXCR: C-X-C chemokine receptor
DEGs: Differentially expressed genes
ECM: Extracellular matrix
hrMIF: Human recombinant MIF
IgG4: Immunoglobulin G4
MAPK: Mitogen-activated protein kinase
MIF: Macrophage Migration Inhibitory Factor
MMPs: Metalloproteinases
PI3K: Phosphoinositide 3-kinase
TCR: T-cell receptor
α-SMA: Alpha-smooth muscle actin
