# Supplementary figures and images for: Macrophage migration inhibitory factor contributes to the pathogenesis of benign lymphoepithelial lesion of the lacrimal gland
Source: Cell Commun Signal. 2018 Oct 22;16:70. doi: 10.1186/s12964-018-0284-4 (PMC6196440; doi:10.1186/s12964-018-0284-4)

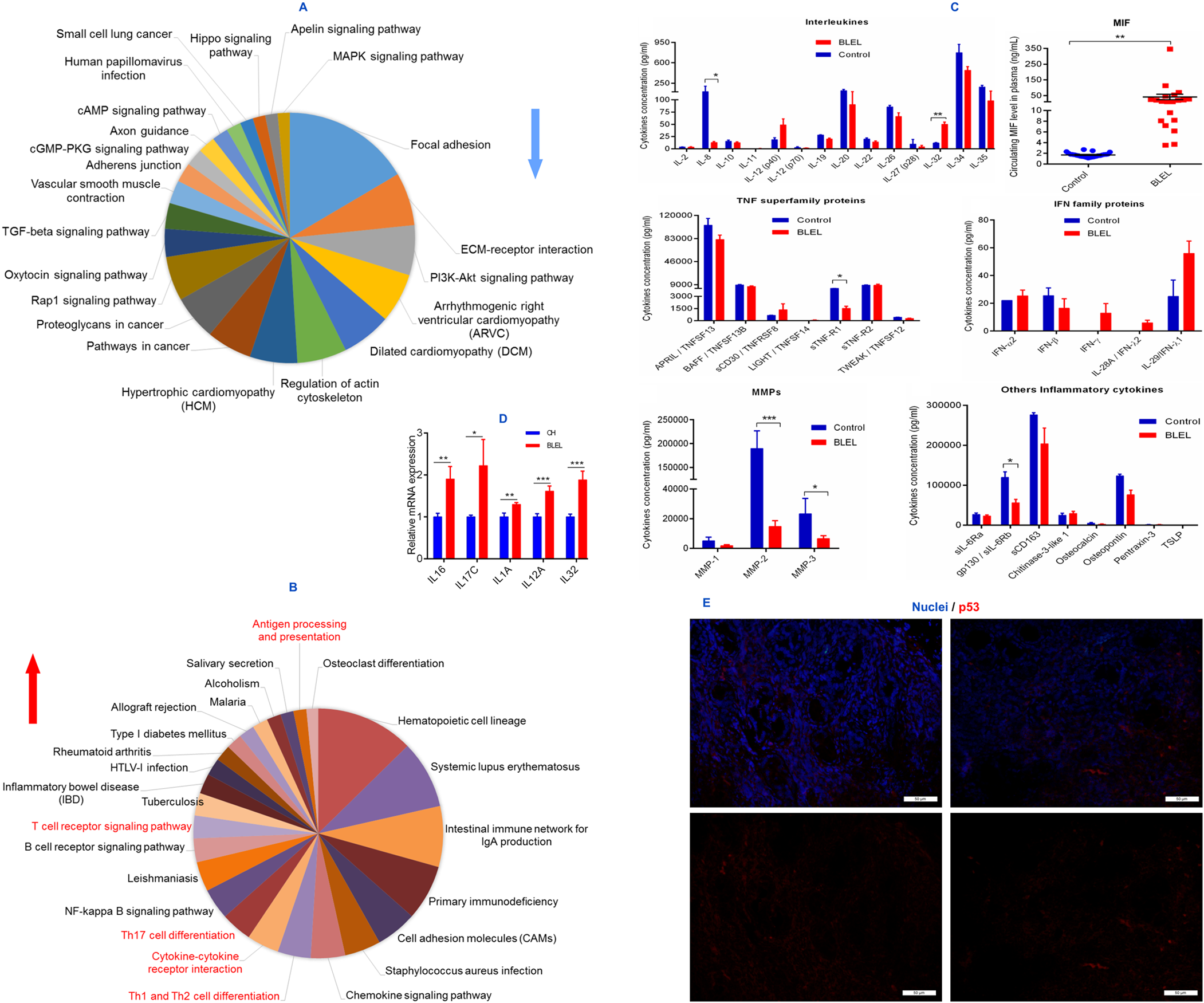

Supplement: Supplementary file 3 — Figure S1. (A & B) KEGG pathways associated to the down-regulated (A) and the up-regulated (B) DEGs. (C) Inflammatory cytokines identified in plasma of BLEL patients. MIF was analyzed from the plasma of 20 BLEL and 25 healthy donors. The other cytokines were assessed from the plasma of 10 BLEL and 4 healthy donors. (D) Microarray data showing the relative mRNA expression of the indicated genes. (E) Fluorescent immunohistochemistry of p53 expression in BLEL tissues. Representative images of fluorescent immunostaining performed on tissue biopsies of 4 CH and 4 BLEL specimens are shown. (A & B) Data were plotted as (−log10) of the corrected p-value. (C & D) Data were plotted as Mean ± SEM, unpaired t-test and multiple t-tests with 1% FDR were used. (E) Original magnification: 40×. (TIF 1592 kb) [file 12964_2018_284_MOESM3_ESM.tif]

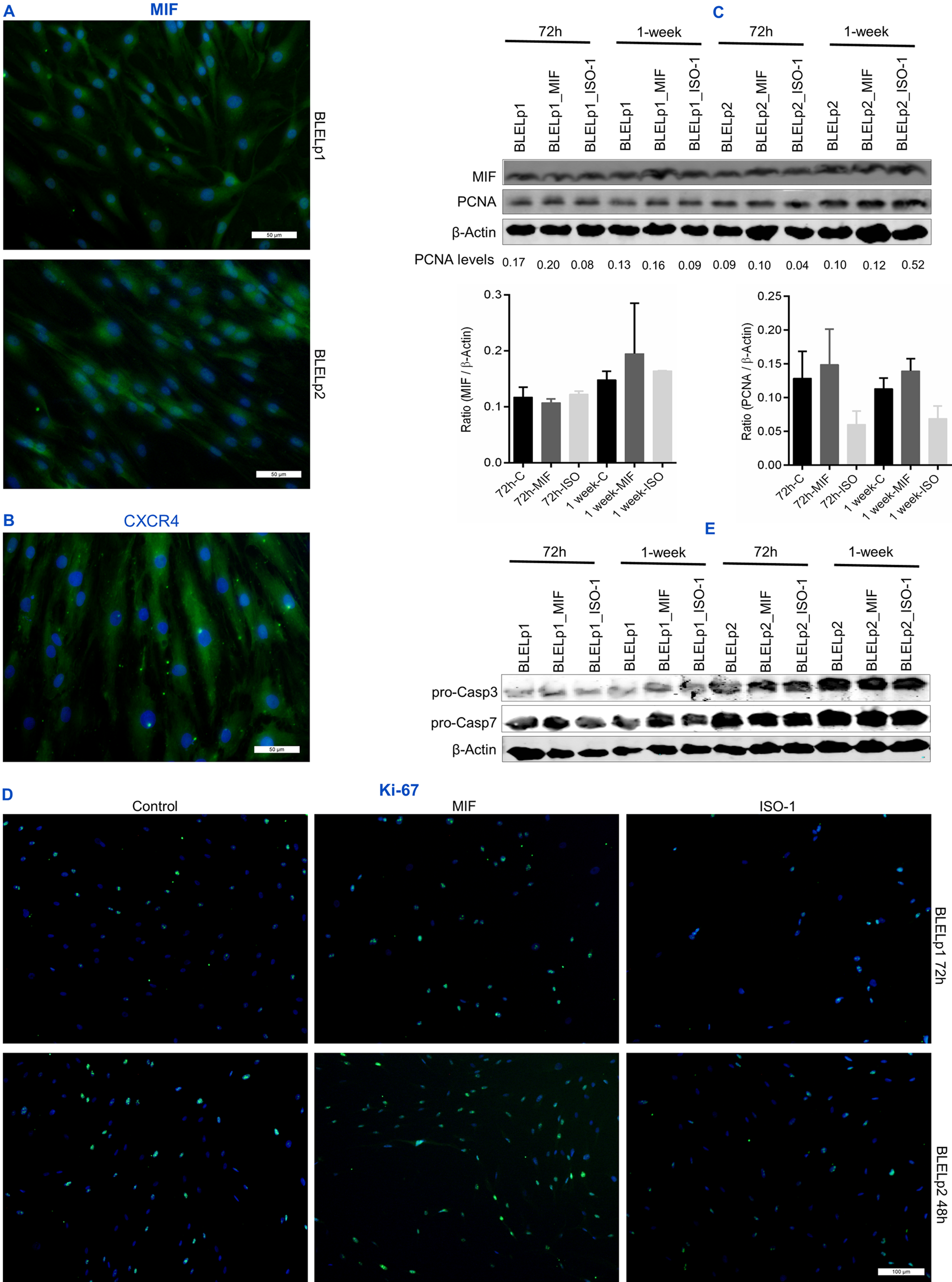

Supplement: Supplementary file 4 — Figure S2. (A&B) Showed representative immunostaining results of MIF and its receptors expression in BLEL primary cells. (C & E) Immunoblotting showing MIF and PCNA (C) and caspases (E) expression in different experimental conditions. BLELp1 and BLELp2 cells were seeded at a density of 106 cells/dish and were treated with MIF (200 ng/ml), ISO-1 (100 μM) or without MIF nor ISO-1 for 72 h or 1 week. (D) Representative images of Ki-67 immunostaining performed in BLELp1 (72 h) and BLELp2 (48 h). Cells from the same suspension were seeded at a density of 2 × 10^4^ cells/well in a 24 wells plate, let be adherent for 24 h and cultured with MIF (200 ng/ml), ISO-1 (100 μM) or without MIF nor ISO-1 at the indicated time point. Original magnification: (A and B) 20× and (D) 10×. (TIF 2580 kb) [file 12964_2018_284_MOESM4_ESM.tif]

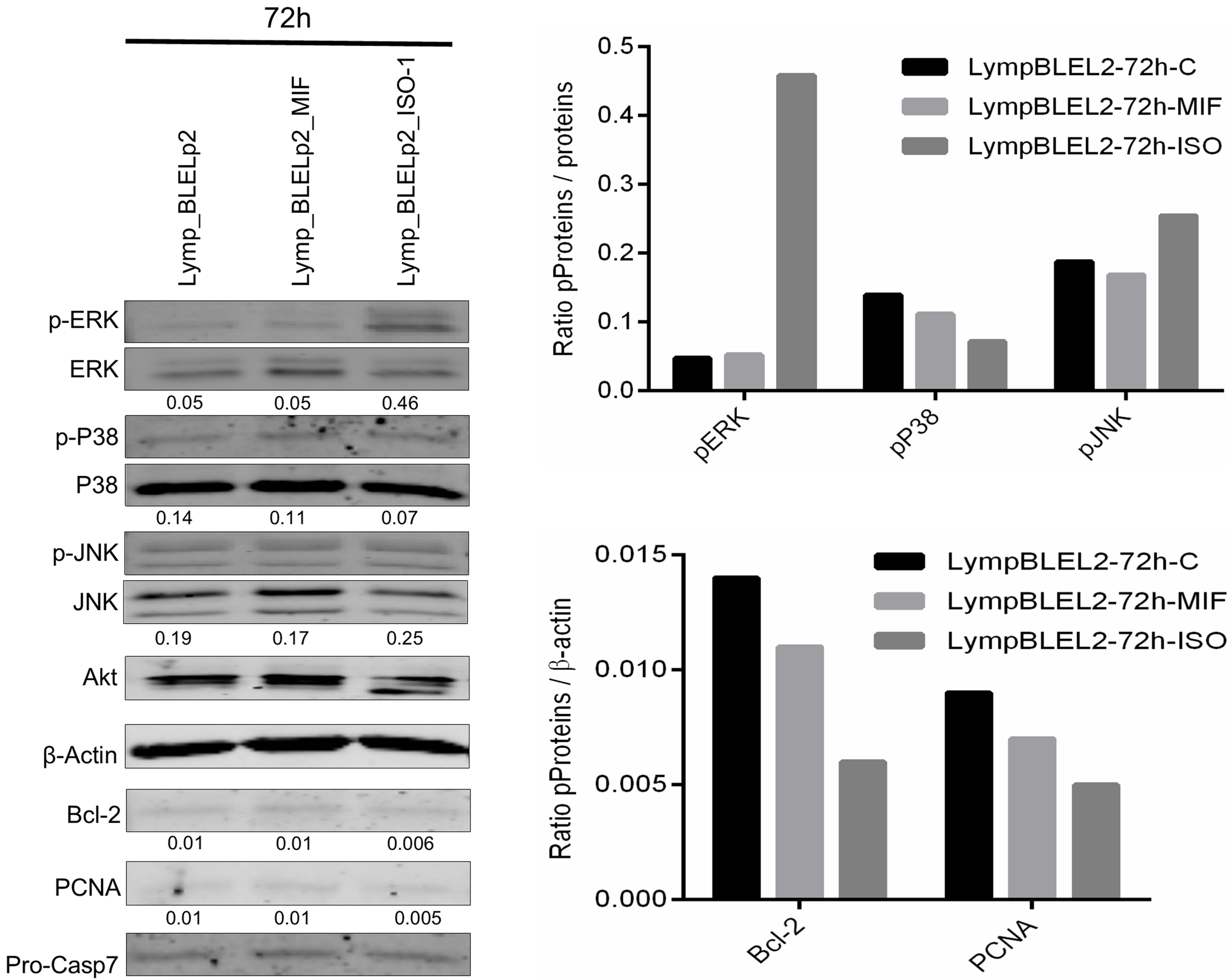

Supplement: Supplementary file 5 — Figure S3. Immunoblotting showing the influence of MIF on the expression of indicated proteins in BLEL tissue-derived lymphocytes (LympBLELp2) following 72 h treatment with MIF (200 ng/ml), ISO-1 (100 μM) or without MIF nor ISO-1. (TIF 568 kb) [file 12964_2018_284_MOESM5_ESM.tif]

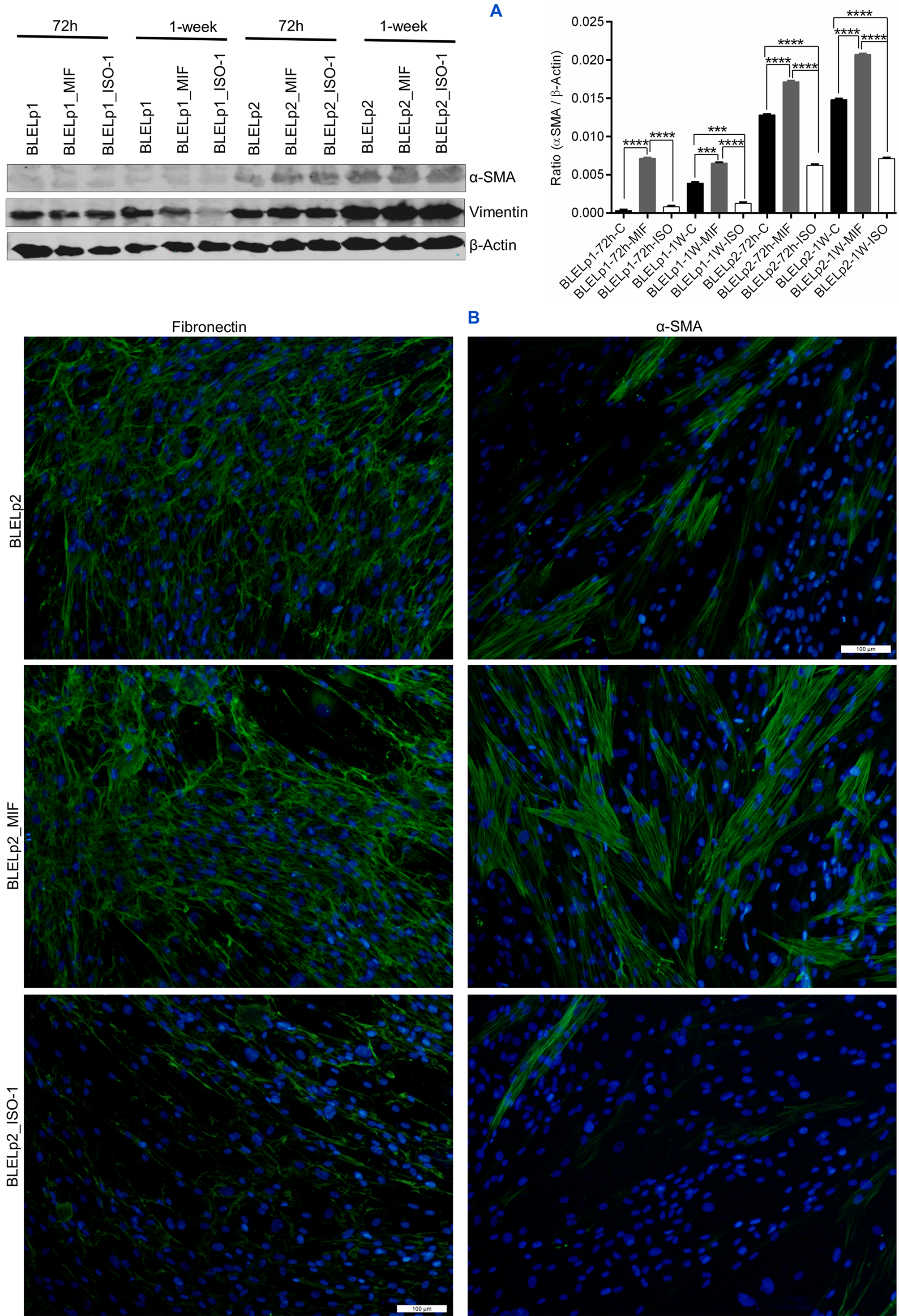

Supplement: Supplementary file 6 — Figure S4. Influence of MIF on αSMA and fibronectin expression. (A) BLELp1 (fibroblast) and BLELp2 (myofibroblast) cells were treated with MIF (200 ng/ml) or ISO-1 (100 μM) or without MIF nor ISO-1 for 72 h and 1 week, (B) BLELp2 (myofibroblast) cells were treated with MIF (200 ng/ml) or ISO-1 (100 μM) or without MIF nor ISO-1 for 1 week. (B) Original magnification: 10×. (TIF 6147 kb) [file 12964_2018_284_MOESM6_ESM.tif]
